# Mapping changes in housing in sub-Saharan Africa from 2000 to 2015

**DOI:** 10.1038/s41586-019-1050-5

**Published:** 2019-03-27

**Authors:** Lucy S. Tusting, Donal Bisanzio, Graham Alabaster, Ewan Cameron, Richard Cibulskis, Michael Davies, Seth Flaxman, Harry S. Gibson, Jakob Knudsen, Charles Mbogo, Fredros O. Okumu, Lorenz von Seidlein, Daniel J. Weiss, Steve W. Lindsay, Peter W. Gething, Samir Bhatt

**Affiliations:** 1 Department of Disease Control, London School of Hygiene & Tropical Medicine, London, UK; 2 RTI International, Washington, DC USA; 3 Division of Epidemiology and Public Health, School of Medicine, University of Nottingham, Nottingham, UK; 4 United Nations Human Settlements Programme, Geneva, Switzerland; 5 Big Data Institute, Nuffield Department of Medicine, University of Oxford, Oxford, UK; 6 Health Metrics and Measurement Cluster, World Health Organization, Geneva, Switzerland; 7 UCL Institute for Environmental Design and Engineering (IEDE), University College London, London, UK; 8 Department of Mathematics and Data Science Institute, Imperial College London, London, UK; 9 School of Architecture, The Royal Danish Academy of Fine Arts, Copenhagen, Denmark; 10 Kenya Medical Research Institute, Kilifi, Kenya; 11 KEMRI-Wellcome Trust Research Program, Nairobi, Kenya; 12 Environmental Health and Ecological Sciences Department, Ifakara Health Institute, Ifakara, Tanzania; 13 School of Public Health, Faculty of Health Sciences, University of the Witwatersrand, Johannesburg, South Africa; 14 Institute of Biodiversity, Animal Health and Comparative Medicine, University of Glasgow, Glasgow, UK; 15 Mahidol-Oxford Tropical Medicine Research Unit (MORU), Faculty of Tropical Medicine, Mahidol University, Bangkok, Thailand; 16 Department of Biosciences, Durham University, Durham, UK; 17 Department of Infectious Disease Epidemiology, Imperial College London, London, UK

**Keywords:** Risk factors, Socioeconomic scenarios, Epidemiology, Developing world, Economics

## Abstract

Access to adequate housing is a fundamental human right, essential to human security, nutrition and health, and a core objective of the United Nations Sustainable Development Goals^1,2^. Globally, the housing need is most acute in Africa, where the population will more than double by 2050. However, existing data on housing quality across Africa are limited primarily to urban areas and are mostly recorded at the national level. Here we quantify changes in housing in sub-Saharan Africa from 2000 to 2015 by combining national survey data within a geostatistical framework. We show a marked transformation of housing in urban and rural sub-Saharan Africa between 2000 and 2015, with the prevalence of improved housing (with improved water and sanitation, sufficient living area and durable construction) doubling from 11% (95% confidence interval, 10–12%) to 23% (21–25%). However, 53 (50–57) million urban Africans (47% (44–50%) of the urban population analysed) were living in unimproved housing in 2015. We provide high-resolution, standardized estimates of housing conditions across sub-Saharan Africa. Our maps provide a baseline for measuring change and a mechanism to guide interventions during the era of the Sustainable Development Goals.

## Abstract (web summary)

The prevalence of improved housing (with improved drinking water and sanitation, sufficient living area and durable construction) in urban and rural sub-Saharan Africa doubled between 2000 and 2015.

## Main

Access to adequate housing and shelter is a fundamental human right, considered central to human wellbeing through the provision of facilities that are essential to security, comfort, health and nutrition. However, major inequalities persist, and a third of the world’s urban population lived in slum conditions in 2014^1^. In response, Sustainable Development Goal 11 aims for universal access to adequate, safe and affordable housing, and to upgrade slums by 2030^2^. This goal builds on Millennium Development Goal 7, which aimed for a substantial improvement in the lives of 100 million people who lived in slums by 2020^3^.

The opportunity and need for better housing is particularly acute in Africa, with its rapidly shifting economic and demographic profile. The continent’s population is the fastest growing in the world and is predicted to increase from 1.2 billion in 2015 to 2.5 billion by 2050 (an addition equivalent to the current population of India)^4^, which will necessitate hundreds of millions of new homes. Alongside increased housing demand, the existing housing stock is steadily transforming—for example, thatch roofs are being replaced by corrugated metal roofs, and mud walls by concrete and brick walls^5^. These changes present a powerful opportunity to improve human wellbeing, and they also demonstrate the urgent need for investment in housing infrastructure to ensure that vulnerable populations are not left behind^6^.

Reliable measurements of house types in Africa are critical for tracking changes and targeting interventions, but existing data on African housing are limited^7^. The primary housing indicator for the United Nations Millennium Development Goals and Sustainable Development Goals is the prevalence of urban slum housing, estimates of which are limited to urban areas only, derived from basic extrapolations from national survey data, restricted to specific years and not standardized across the continent at any subnational scale^8,9^. Other detailed records of African housing conditions are focused on housing costs and finance^10^. Here we conduct a standardized analysis using a geospatial framework to quantify the changing profile of housing in urban and rural sub-Saharan Africa during the era of the Millennium Development Goals. We show that African housing underwent a marked change between 2000 and 2015, but unimproved housing persists.

**Extended Data Fig. 1:**
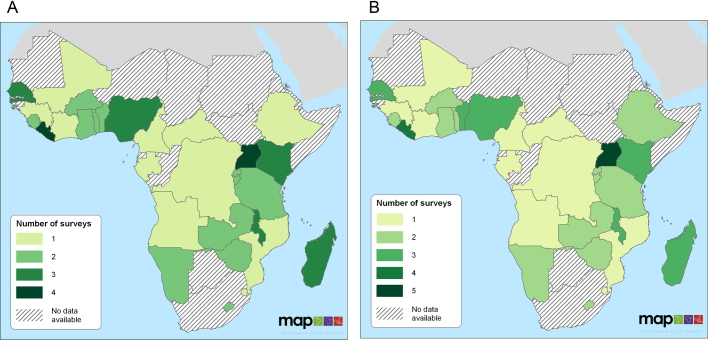
Availability of national survey data for the period 1990–2016 for the variables that are required to determine house construction materials and house type in sub-Saharan Africa. **a**, Availability of surveys for the determination of house construction materials. **b**, Availability of surveys for the determination of house type. Maps were produced using ArcGIS.

To quantify changes in housing across sub-Saharan Africa, we leveraged 62 georeferenced national household surveys, representing 661,945 unique households in 31 countries (Extended Data Fig. 1). We designed a geostatistical regression model to map house construction materials and overall house type at 5 × 5-km^2^ resolution across sub-Saharan Africa. We categorized house construction materials into a binary variable that compared houses built from finished materials (for example, parquet, vinyl, tiled, cement or carpet flooring) to those built from natural or unfinished materials (for example, earth, sand, dung or palm flooring) (Extended Data Table 1). We based our categorization of house type on the Millennium Development Goal and Sustainable Development Goal definition of slum housing. We considered ‘unimproved’ housing to have at least one of four characteristics: (1) unimproved water supply; (2) unimproved sanitation; (3) more than three people per bedroom; and (4) house made of natural or unfinished materials (Supplementary Methods). We considered houses that had none of these characteristics as ‘improved’.

**Extended Data Fig. 2:**
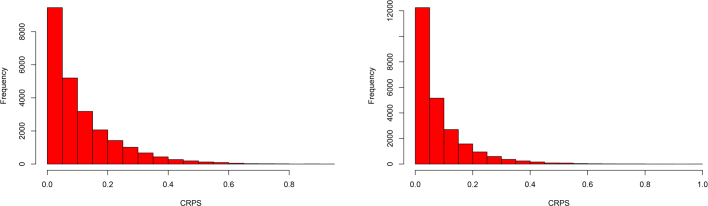
The continuous ranked probability scores for the models of house type and house construction material. Continuous ranked probability scores (CRPS) are shown for the house type model (left; mean = 0.11) and the house construction material model (right; mean = 0.08). Both distributions indicate well-calibrated confidence intervals with clustering towards zero; that is, a low error with regards to the distribution of the prediction.

The independent variables (covariates) used in our model were aridity^11^, urbanicity^12^, accessibility^13^, travel friction^13^, night-time lights^14^ and irrigation^15^, which are commonly used in Africa-focused geostatistical models^16^; we also included space and time to account for autocorrelated residual effects. Our geostatistical model utilizes the random Fourier feature approach^17^ in which a nonlinear, interacting function is defined through high-dimensional feature spaces computed in explicit form in a feature map. The feature map that characterizes this relationship $\left(\varphi :{\mathscr{X}}\to {\mathscr{H}}\right)$ induces a measure of similarity—the kernel function $\left(k:{\mathscr{X}}\times {\mathscr{X}}\to {\mathbb{R}}\right)$—such that $k\left({x}_{i},{x}_{j}\right)={\left(\varphi \left({x}_{i}\right),\varphi \left({x}_{j}\right)\right)}_{{\mathscr{H}}}$ . Expanding on a previous study^17^, our feature map takes the Fourier spectral form $z(x|\omega )={[{\rm{c}}{\rm{o}}{\rm{s}}({x}^{T}\omega ){\rm{s}}{\rm{i}}{\rm{n}}({x}^{T}\omega )]}^{T}$such that $k\left({x}_{i},{x}_{j}|\theta \right)\approx \frac{{\sigma }^{2}}{{N}_{{\rm{feat}}}}{\sum }_{r=1}^{M}z{\left({x}_{i}|{\omega }_{r}\right)}^{T}z({x}_{j}|{\omega }_{r})$   , with spectral measure *ω*_*r*_. Rather than assuming a specific spectral distribution *ω*_*r*_, we obtain this Lebesgue measure directly from the data^18^. Given a response variable (for example, wall material), we used a beta-binomial likelihood function *p*([*y*^+^, *y*^total^] | *x*, *ω*, *ϕ*) = BetaBinomial(*z*(*x*|*ω*), *ϕ*) that enabled overdispersion in the data while simultaneously accounting for sample size variation (from *y*^total^*y*^total^). We performed regularization using dropout^19^. Approximate posterior confidence intervals were estimated using the weighted likelihood bootstrap^20^. Fitting was performed using ADAM stochastic gradient descent^21^. Cross-validation was performed to check for model fit and to assess the predictive accuracy of the model (Supplementary Text, Extended Data Figs. 2, 9). Population-weighted prevalences of people living in different house types in urban and rural areas were calculated using yearly population data from the WorldPop project^22^ and a static urban–rural definition from the Global Urban Footprint project^23^.

**Fig. 1:**
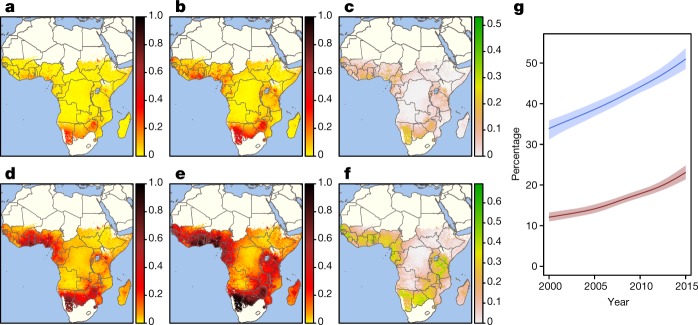
Changes in housing in sub-Saharan Africa between 2000 and 2015. **a**, Prevalence of improved housing across sub-Saharan Africa in 2000 predicted at 5 × 5-km^2^ resolution. **b**, Prevalence of improved housing in 2015 predicted at 5 × 5-km^2^ resolution. **c**, Absolute difference in the prevalence of improved housing in 2000 and 2015. **d**, Prevalence of houses built with finished materials in 2000 predicted at 5 × 5-km^2^ resolution. **e**, Prevalence of houses built with finished materials in 2015 predicted at 5 × 5-km^2^ resolution. **f**, Absolute difference in prevalence of houses built with finished materials in 2000 and 2015. **g**, Increase in prevalence of improved housing (red line; shading, 95% confidence intervals) and housing built with finished materials (blue line) from 2000 to 2015. Results are derived from a geospatial model fitted to 62 surveys that represent 661,945 households (house construction materials) and 59 surveys that represent 629,298 households (house type). Houses were classified as improved if they had all of the following characteristics: improved water supply, improved sanitation, three or fewer people per bedroom and house made of finished materials (Extended Data Table 1 and Supplementary Methods). Maps were produced using the raster package (version 2.6-7) in R. The images were plotted using the rasterVis package (version 3.4).

**Fig. 2:**
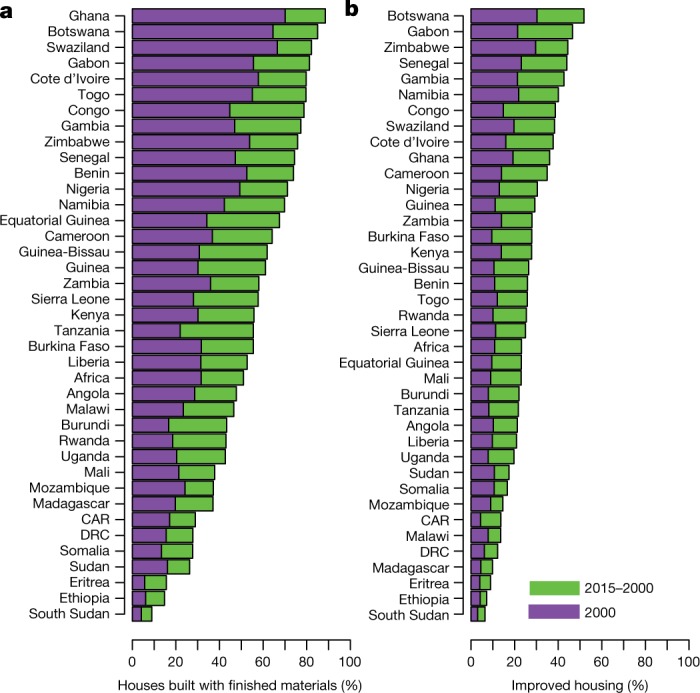
National-level changes in housing between 2000 and 2015. **a**, **b**, Plots show predicted population-weighted mean prevalence of houses built with finished materials (**a**) and improved housing (**b**). Bars represent each country in 2000 (purple) and 2015 (purple and green combined). Houses were classified as improved if they had all of the following characteristics: improved water supply, improved sanitation, three or fewer people per bedroom and house made of finished materials (Extended Data Table 1 and Supplementary Methods). CAR, Central African Republic; Congo, Republic of the Congo; DRC, Democratic Republic of the Congo.

**Extended Data Fig. 3:**
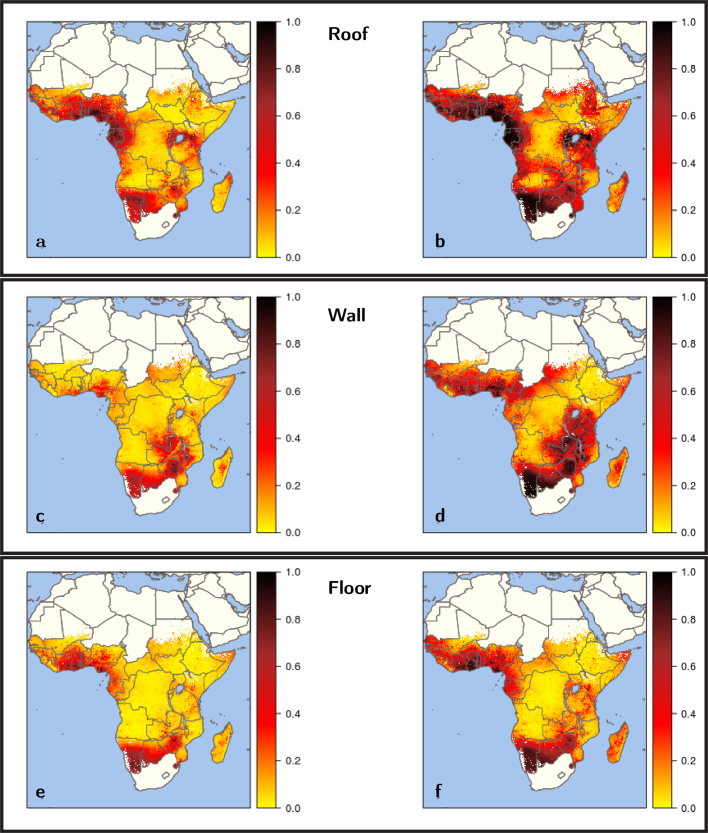
Prevalence of main roof, wall and floor material of houses in sub-Saharan Africa in 2000 and 2015. **a**, Prevalence of houses built with finished (versus natural or unfinished) roof material in 2000 predicted at 5 × 5-km^2^ resolution. **b**, Prevalence of houses built with finished (versus natural or unfinished) roof material in 2015 predicted at 5 × 5-km^2^ resolution. **c**, Prevalence of houses built with finished (versus natural or unfinished) wall material in 2000 predicted at 5 × 5-km^2^ resolution. **d**, Prevalence of houses built with finished (versus natural or unfinished) wall material in 2015 predicted at 5 × 5-km^2^ resolution. **e**, Prevalence of houses built with finished (versus natural or unfinished) floor material in 2000 predicted at 5 × 5-km^2^ resolution. **f**, Prevalence of houses built with finished (versus natural or unfinished) floor material in 2015 predicted at 5 × 5-km^2^ resolution. Results are derived from a geospatial model fitted to 66 surveys for roof material, 96 surveys for floor material and 62 surveys for wall material. Maps were produced using the raster package (v.2.6-7) in R. The images were plotted using the rasterVis package (v.3.4).

Our analysis revealed a marked transformation of housing in sub-Saharan Africa from 2000 to 2015. Across all sub-Saharan countries (excluding South Africa, Comoros and desert areas), the prevalence of houses that were built with finished materials increased from 32% (29–33%) in 2000 to 51% (49–54%) in 2015 (Table 1 and Figs. 1, 2). Our analysis suggests a widespread pattern of incremental modifications to the roof, then the walls and finally to the floor of houses (Extended Data Fig. 3). Overall, the predicted prevalence of improved housing (with improved water and sanitation, sufficient living area and durable construction) doubled from 11% (10–12%) in 2000 to 23% (21–25%) in 2015 (Table 1), with prevalences ranging from 5% (5–6%) in rural Ethiopia to 76% (71–80%) in urban Zimbabwe in 2015 (Supplementary Table 1). Between 2000 and 2015, 134 (118–147) million Africans in the analysed countries gained access to improved housing. However, unacceptable inequalities persist, with 53 (50–57) million urban inhabitants (47% (44–50%) of the total urban population of sub-Saharan Africa analysed) and 595 (585–607) million rural inhabitants (82% (80–83%) of the rural population) living in unimproved housing in 2015.

**Table 1:** Changes in house types across sub-Saharan Africa from 2000 to 2015

Population	Number of people		Prevalence (%) of people living in houses built with finished materials (95% CI)		Prevalence (%) of people living in improved housing (95% CI)	
	2000	2015	2000	2015	2000	2015
All	562,367,947	842,438,941	31.6 (28.8–33.4)	51.0 (48.5–53.6)	10.9 (9.9–12.0)	23.2 (21.4–24.8)
Urban	61,501,994	113,530,870	80.3 (76.8–82.5)	91.5 (90.6–92.3)	32.3 (29.2–35.4)	53.2 (50.1–56.1)
Rural	500,865,953	728,908,071	25.6 (22.8–27.5)	44.7 (41.9–47.6)	8.2 (7.5–9.1)	18.4 (16.7–19.8)

Predictions represent all countries in sub-Saharan Africa excluding South Africa, Comoros and desert areas. Houses were considered to be built with finished materials if at least two out of three parts of the structure (walls, roof and floor) were made of finished materials rather than natural or unfinished materials (Supplementary Text). Houses were considered to be ‘improved’ if they had all of the following characteristics: improved water supply, improved sanitation, three or fewer people per bedroom and house made of finished materials (Supplementary Information). CI, confidence interval.

**Fig. 3:**
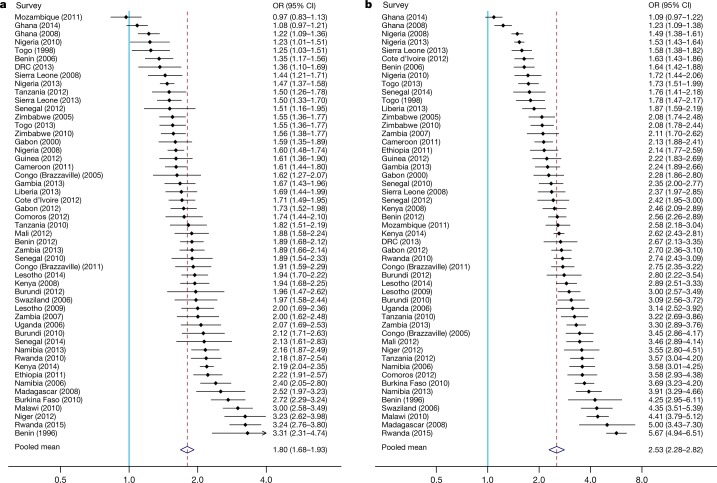
Association between house type, education and household wealth. **a**, Association between house type and education level. The pooled increase in odds of living in an improved house when the household head reported having completed more than primary education, compared to having primary education or less, is shown by the diamond and dashed red vertical line. The solid blue vertical line represents the null value (no difference between groups). Odds ratios (OR) are adjusted for wealth index, age of the household head and geographical cluster. Error bars show 95% confidence intervals. **b**, Association between house type and household wealth. The pooled increase in odds of living in an improved house among households in the upper 75% wealth quartile compared to all other households is shown. Odds ratios are adjusted for education level, age of the household head and geographical cluster. Data are from 48 Demographic and Health Surveys, two Malaria Indicator Surveys and one AIDS Indicator Survey, conducted between 1996 and 2015 (Supplementary Table 2).

**Extended Data Fig. 4:**
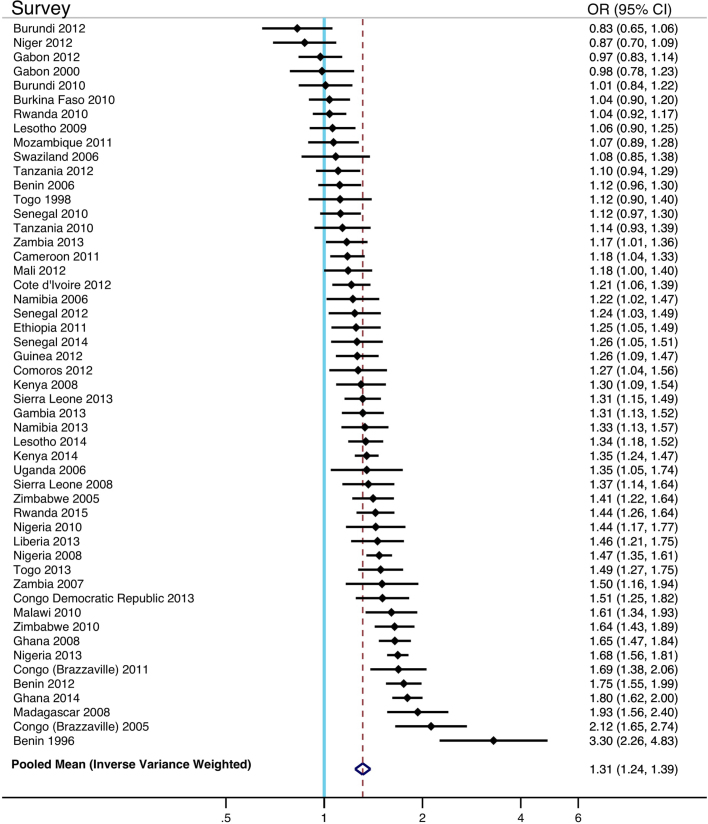
Association between house type and age of the household head. The pooled increase in odds of living in an improved house when the age of the household head is over 55 years, compared to 55 years or less, is shown to the right of the vertical line representing the null value (no difference between groups). Odds ratios are adjusted for wealth index, education level of the household head and geographical cluster. Error bars show 95% confidence intervals. Data are from 48 DHS, 2 MIS and 1 AIS conducted between 1996 and 2015 (Supplementary Table 2).

**Extended Data Fig. 5:**
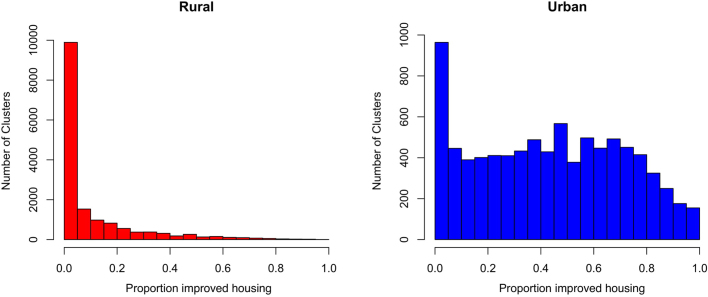
Prevalence of improved housing in rural and urban survey clusters. Data are from 59 national household surveys from 31 countries in sub-Saharan Africa conducted between 1994 and 2015.

**Extended Data Fig. 6:**
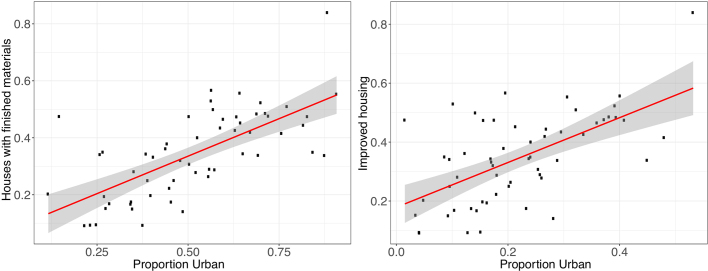
Prevalence of house types in relation to survey-level prevalence of urban clusters. Left, house construction materials (adjusted *R*^2^ = 0.46, *P* < 0.001). Right, house type (adjusted *R*^2^ = 0.35, *P* < 0.001). Points represent each national survey included in the analysis.

To examine the links between housing and socioeconomic factors, we quantified the association between house type and household characteristics in 51 national surveys, representing 588,892 households (Supplementary Table 2). For each survey, controlling for cluster-level variation, we jointly estimated the odds of improved housing in relation to education level of the household head, household wealth and age of the household head. We found that the odds of improved housing were 80% higher in more educated households (adjusted odds ratio, 1.80; 95% confidence interval, 1.68–1.93; *P* < 0.001; Fig. 3a), more than double in the wealthiest households (adjusted odds ratio, 2.53; 95% confidence interval, 2.28–2.82; *P* < 0.001; Fig. 3b) and 31% higher with increased age of the household head (adjusted odds ratio, 1.31; 95% confidence interval, 1.24–1.39; Extended Data Fig. 4). We also observed a higher prevalence of improved housing in urban survey clusters than rural survey clusters (Extended Data Fig. 5). Across all surveys, a 10% increase in the prevalence of urban clusters (including small rural towns that have grown from villages and account for much of sub-Saharan Africa’s urban growth) was associated with a 7.5% increase in improved housing (Extended Data Fig. 6).

Here we quantified housing conditions across urban and rural sub-Saharan Africa during the era of the Millenium Development Goals, and provided a detailed baseline measurement for the Sustainable Development Goals. By applying a geospatial approach to empirical observations, we have built considerably on existing measurements of African housing, which are limited to urban areas, not standardized at any subnational scale and are derived from more simplistic extrapolations from survey data. We show that the prevalence of improved housing (defined as housing with improved water and sanitation, sufficient living area and durable construction) doubled during 2000–2015, but that an unacceptably large proportion of people still live in unimproved housing in urban areas.

**Extended Data Fig. 7:**
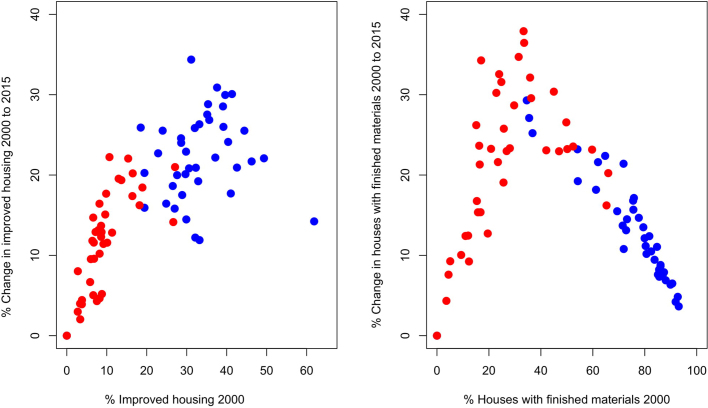
Changes in housing in sub-Saharan Africa from 2000 to 2015 relative to the 2000 baseline. Points represent countries stratified by urban (blue) and rural (red) areas.

Our findings are consistent with continent-wide changes to African housing being driven by economic growth^24^. Increasing household spending is likely to have led people to invest more in their homes and, indeed, we found a clear increase in the prevalence of houses built with finished materials since 2000. Furthermore, house types changed the most in countries with the highest baseline prevalence of improved housing (Extended Data Fig. 7) and house type was clearly associated at the household level with education, wealth and age of the household head. In urban areas, the changes may also have been driven by a lack of traditional materials and the commodification of housing. In the future, continued population and urban growth in sub-Saharan Africa may help to sustain housing demand and incremental housing changes. In turn, ‘healthy urbanization’ has been recognized as important for maintaining economic productivity and growth^24^.

**Extended Data Fig. 8:**
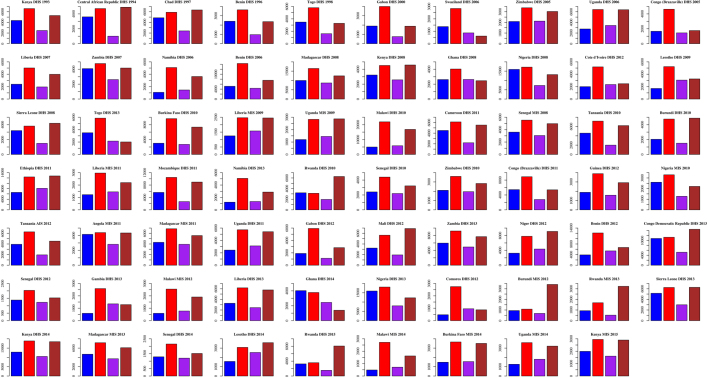
Number of households per survey that lack the characteristics of improved houses. Characteristics shown are improved water source (blue), improved sanitation facilities (red), sufficient living area (purple) and finished house construction materials (brown). Data are from 1 AIS, 15 MIS and 53 DHS that had data on all four of these variables, conducted between 1993 and 2015. Out of a total of 69 surveys, households most frequently lacked improved sanitation facilities (52 surveys; 75%) and finished materials (16 surveys; 23%).

Our study has important implications for international goals, which have sought to address housing inequalities by achieving a substantial improvement in the lives of 100 million people living in slums by 2020^3^ and universal access to adequate, safe and affordable housing by 2030^2^. We show a considerable reduction in the prevalence of urban unimproved housing across sub-Saharan Africa from 68% (65–71%) in 2000 to 47% (44–50%) in 2015, similar to the equivalent estimates from the United Nations of 65% in 2000 and 55% in 2014^25^. However, nearly half of Africa’s urban population still lives in unimproved conditions, which is partly explained by widespread unimproved sanitation—the most common housing deprivation in 75% (52 out of 69) of surveys analysed (Extended Data Fig. 8). These findings highlight the urgent need for governments to improve water and sanitation infrastructure as households continue to spend individually on their homes.

Housing is a central pillar of human security and wellbeing and is increasingly vital in the context of Africa’s urbanization and population growth. For example, house design is integral to Sustainable Development Goal 3 through a myriad of associated health outcomes, including mental health, respiratory disease, soil-transmitted helminths, diarrhoeal disease, leishmaniasis and malaria^26–28^. As towns and cities in sub-Saharan Africa grow, rapid development of luxury housing in major urban centres is occurring alongside the expansion of informal settlements that lack basic infrastructure. In addition, formal housing investment typically lags behind urbanization and the continent’s major urban growth is concentrated in smaller urban centres that have limited capacity to organize construction^6^. Addressing the housing needs of a growing population is key for sustainable urban development and the health and wellbeing of millions of Africans^29^, and will facilitate faster attainment of the Sustainable Development Goals. Our maps provide a critical mechanism to guide intervention and the measurement of change.

## Methods

### Data reporting

No statistical methods were used to predetermine sample size. The experiments were not randomized and the investigators were not blinded to allocation during experiments and outcome assessment.

### Data sources

Data were sourced from Demographic and Health Surveys (DHS), Malaria Indicator Surveys (MIS) and AIDS Indicator Surveys (AIS), which are cross-sectional surveys designed to collect nationally representative health and sociodemographic data, typically at 3–5-year intervals^30,31^. Surveys are administered using a stratified two-stage cluster design in which primary sampling units are randomly selected from census data and households are randomly selected within primary sampling units from an updated enumeration list. Raw DHS datasets were downloaded from www.dhsprogram.com and data were directly categorized into the household-level variables included in the analysis, using the definitions below.

### Definition of house type

We explored two aspects of housing: (1) house construction materials; and (2) overall house type (Extended Data Table 1). DHS and MIS record the main materials used for the roof, walls and/or floor^32^ and categorize these as ‘natural’, ‘rudimentary’ or ‘finished’ (for example, finished floor materials include parquet, vinyl, ceramic tiles, cement and carpet, whereas natural or rudimentary floor materials include earth, sand, dung, wood planks, palm and bamboo^33^). We classified houses as ‘built from finished materials’ if at least two out of three of the materials for the walls, roof and floor were finished and as ‘built from natural or unfinished materials’ if this criterion was not met. We based our categorization of house type on the definition of slum housing^3^ of the United Nations Millennium Development Goals and Sustainable Development Goals. ‘Unimproved’ houses were considered to have at least one of four characteristics: (1) unimproved water supply (as defined by the World Health Organization Joint Monitoring Programme (WHO JMP)^34^ (Supplementary Table 3)); (2) unimproved sanitation (as defined by WHO JMP^34^); (3) more than three people per bedroom; and (4) house made of natural or unfinished material. Houses that had none of these characteristics were considered as ‘improved’. Following the United Nations protocol, we excluded a fifth characteristic of unimproved housing from our definition (insecurity of tenure) due to the lack of internationally comparable data^3^.

### Predicting changes in housing from 2000 to 2015

We included in the analysis all georeferenced surveys (that is, latitude and longitude were available for each geographical cluster) with data on water supply, sanitation facilities, number of household members, number of bedrooms and main material of the roof, walls and floor (Extended Data Fig. 1 and Supplementary Table 4). We designed a geostatistical regression model to map (1) main material of the roof, walls and floor; and (2) overall house type at 5 × 5-km^2^ resolution across sub-Saharan Africa. The independent variables (covariates) used in our model were aridity index^11^, degree of urbanicity^12^, accessibility to large cities^13^, travel friction surface^13^, night-time lights^14^ and irrigation^15^. These independent variables were chosen as close proxies for factors that affect house type, such as poverty, development, urbanization, transport access and population density. We also included spatial coordinates and time, to account for spatio-temporally autocorrelated residual effects. Our geostatistical model utilizes the random Fourier feature approach^17^ for which a nonlinear, interacting function is defined through high-dimensional feature spaces computed in explicit form through a feature map. This approach approximates a kernel function through an explicit rather than implicit map. The feature map associates a kernel function $k:{\mathscr{X}}\times {\mathscr{X}}\to {\mathbb{R}}$, which is defined on an input domain ${\mathscr{X}}\in \left\{{x}_{1},..,{x}_{d}\right\}\in {{\mathbb{R}}}^{d}$ such that $k\left({x}_{i},{x}_{j}\right)={\left(\varphi \left({x}_{i}\right),\varphi \left({x}_{j}\right)\right)}_{{\mathscr{H}}}$ where $\varphi :{\mathscr{X}}\to {\mathscr{H}}$ is the feature map that associates kernel *k* with an embedding of the input space into a reproducing kernel Hilbert space ${\mathscr{H}}$. On the basis of a previous study^17^, our feature map takes the form $z(x|\omega )={[{\rm{c}}{\rm{o}}{\rm{s}}({x}^{T}\omega ){\rm{s}}{\rm{i}}{\rm{n}}({x}^{T}\omega )]}^{T}$ such that $k\left({x}_{i},{x}_{j}|\theta \right)\approx \frac{{\sigma }^{2}}{{N}_{{\rm{feat}}}}{\sum }_{r=1}^{M}z{\left({x}_{i}|{\omega }_{r}\right)}^{T}z({x}_{j}|{\omega }_{r})$ with a given spectral measure *ω*_*r*_. Rather than assuming a spectral distribution in which *ω*_*r*_ is associated with a given kernel (for example, Student’s *t* for the Matérn kernel), we obtained this distribution (empirical Lebesgue measure) directly from the data^18^. Given a response variable (for example, wall type), we used a beta-binomial likelihood function $p(y|x,\omega ,\varphi )={\rm{BetaBinomial}}\left(z\left(x|\omega \right),\varphi \right)$ to perform inference, allowing for overdispersion and sample size effects in the data. We performed regularization using dropout^19^. Dropout was chosen over standard ${{\ell }}_{1},{{\ell }}_{2}$ penalties owing to ease of implementation, parameter tuning and superior cross-validation performance. We included length scale parameters for each covariate dimension by shrinking or expanding the empirical measure *ω*. These unconstrained length scale parameters were passed through a reticulated linear function to ensure positivity and sparse covariate selection (known as automatic relevance determination). Fitting was performed using ADAM stochastic gradient descent^21^ with GPU TensorFlow. Validation was carried out to check for appropriate model fit and to assess the predictive accuracy of the model. The predictive performance of the model at the pixel level and administrative division level 1 was assessed via out-of-sample validation. The dropout hyperparameter was selected by random search. The robustness of this hyperparameter was double-checked by implementing automatic selection of the hyperparameter through concrete dropout^35^. Confidence intervals and uncertainty were estimated using the weighted likelihood bootstrap, a method that generates samples from an approximate Bayesian posterior of a parametric model^20^. In brief, this approach involved repeated fitting of the model while weighting the likelihood with uniform Dirichlet weights—that is, $p{\left(y|x,\omega \right)}^{w}$ where $w \sim {\rm{Dirichlet}}\left(1,1,\ldots ,n\right)$. We performed this procedure 100 times to obtain 100 realizations of the model and subsequent surfaces. Population-weighted prevalences of people living in different house types in urban and rural areas were calculated using yearly population data from the WorldPop project^22^ and using a static urban-rural definition from the Global Urban Footprint project^23^.

### Model performance

**Extended Data Fig. 9:**
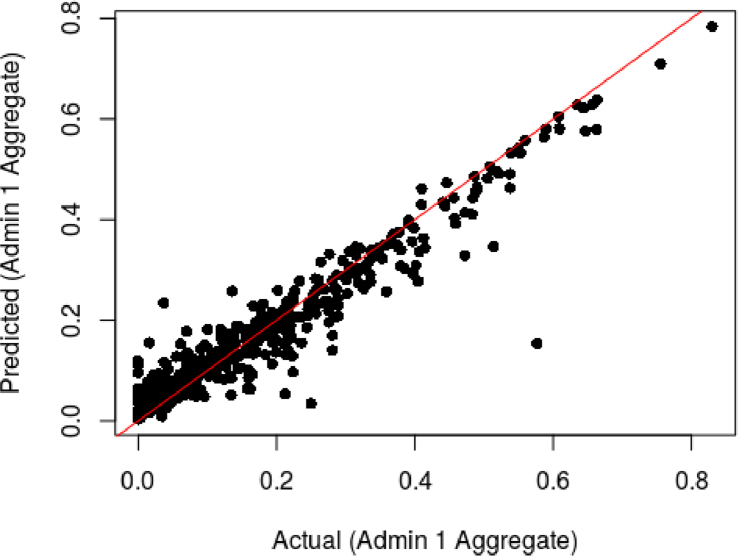
Observed versus predicted prevalence of improved housing aggregated to district level (administrative division 1 level). Fit predictions for both observed and predicted prevalence were aggregated to the district level and plotted.

The predictive performance of both the improved housing and finished materials models at pixel level was assessed through out-of-sample validation. In this validation scheme, the full dataset was randomly partitioned such that 75% of the dataset was used to fit the model and the remaining 25% was used to test the model predictive performance. This scheme was repeated 10 times and the scores were averaged. The mean squared error^36^ and the correlation coefficient were used to evaluate model performance. We evaluated the model at both the pixel level and the aggregate survey level. The latter was included to give an indication of how well the model predicts the survey as a whole. The predictive scores (Supplementary Table 6 and Extended Data Fig. 9) indicate excellent model performance, comparable to those from widely used, established models^16^. In addition, as pixel-level estimates we performed additional cross-validation analyses in which entire administrative divisions of data were held out. This allowed us to compare our predictions with those from surveys at a resolution at which the surveys are reliable. The results from this validation support the conclusions from our primary analysis (Supplementary Table 7). Confidence intervals were evaluated by the widely used continuous ranked probability score, a score that generalizes the mean absolute error to probability distributions^36^. The continuous ranked probability scores show suitable credible intervals with most scores clustering towards zero.

### Household-level association between house type and socioeconomic factors

Binary variables for education, wealth and age of the household head were created at the household level. National surveys record the highest level of education of the household head and we compared more than primary education with primary education or less. DHS and MIS household wealth index scores are developed using principal component analyses that typically include variables that describe durable asset ownership, access to utilities and infrastructure and house construction materials^37^. To enable estimation of the association between house type and wealth, we constructed a new asset-based wealth index for each survey that excluded variables related to house construction. For each household survey, we applied inclusion criteria^27^ of (1) fewer than 10% missing values; and (2) frequency values between 5% and 95% for the following set of assets: (a) car; (b) motorboat; (c) scooter; (d) cart; (e) bicycle; (f) television; (g) refrigerator; (h) radio; (i) watch; (j) mobile telephone; (k) landline telephone; and (l) electrification of the household. We tested several dimensionality reduction algorithms, with the goal of condensing these twelve assets into a single dimensional index. We tested ISOMAP^38^, kernel principal component analysis (PCA)^39^, *t*-distributed stochastic neighbour embedding^40^ and linear PCA^41^. We found minimal differences between algorithms and therefore opted for linear PCA. Using the first principal component, we created a binary wealth variable comparing households belonging to the upper 75th wealth quartile with all other households.

We examined the association between house type and education level of the household head, household wealth and age of the household head in 51 cross-sectional household surveys, representing 588,892 households. For each survey, we jointly estimated the odds of improved housing in relation to whether the household head completed at least primary education, whether a household belonged to the upper 75% wealth quartile and whether the household head was aged over 55 years (which is approximately the upper 75% age quartile). We performed conditional logistic regression to allow these associations to be estimated within geographical cluster in order to eliminate confounding due to inter-cluster variation in urbanicity, regional wealth, climate and other survey design factors. Individual survey odds ratios were combined to determine a summary odds ratio for all surveys using random effects meta-analysis. Individual and summary odds ratios were displayed in forest plots.

### Reporting summary

Further information on research design is available in the Nature Research Reporting Summary linked to this paper.

### Code availability

Analysis code is available via codeshare: https://codeshare.io/2pm4Px.

## Online content

Any methods, additional references, Nature Research reporting summaries, source data, statements of data availability and associated accession codes are available at 10.1038/s41586-019-1050-5.

### Supplementary information

Supplementary Information This file contains Supplementary Methods and Supplementary Tables 1–7. The Supplementary Methods contain additional details of the house type classification. Seven display items (Supplementary Tables) provide details of the datasets analysed, house type classifications, model performance and detailed predictions of house type prevalence by country for 2000 and 2015.

Reporting Summary

## Figures and Tables

**Extended Data Table 1:**
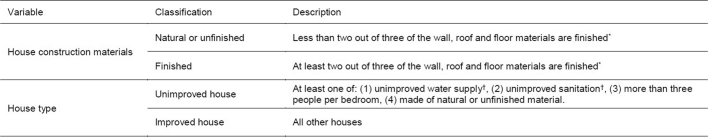
Definition of house type variables

*Main material of the wall, roof and floor are recorded in national surveys (for example, DHS) and pre-categorized by the local investigators as ‘natural’, ‘rudimentary’ or ‘finished’ (Supplementary Text and Supplementary Table 5).

^†^Water supply and sanitation facilities were classified using World Health Organization Joint Monitoring Programme criteria (Supplementary Table 3).

## Data Availability

All data are available to download free of charge by registered users from the DHS Program. Registration is available at https://dhsprogram.com/data/new-user-registration.cfm and data may be downloaded at https://dhsprogram.com/data/dataset_admin/download-manager.cfm. Full instructions to access the datasets are available at https://dhsprogram.com/data/Using-DataSets-for-Analysis.cfm. The housing maps are available for visualization and/or download at https://map.ox.ac.uk/research-project/housing_in_africa/.
