# Supplementary material for: Mapping changes in housing in sub-Saharan Africa from 2000 to 2015
Source: Nature. 2019 Mar 27;568(7752):391–4. doi: 10.1038/s41586-019-1050-5 (PMC6784864; doi:10.1038/s41586-019-1050-5)
Supplement: Supplementary file 1 — This file contains Supplementary Methods and Supplementary Tables 1–7. The Supplementary Methods contain additional details of the house type classification. Seven display items (Supplementary Tables) provide details of the datasets analysed, house type classifications, model performance and detailed predictions of house type prevalence by country for 2000 and 2015. [file 41586_2019_1050_MOESM1_ESM.pdf]

nature

SUPPLEMENTARY INFORMATION

https://doi.org/10.1038/s41586-019-1050-5

In the format provided by the authors and unedited.

Mapping changes in housing in sub-Saharan Africa from 2000 to 2015

Lucy S. Tusting[1*], Donal Bisanzio[2,3], Graham Alabaster[4], Ewan Cameron[5], Richard Cibulskis[6], Michael Davies[7], Seth Flaxman[8], Harry S. Gibson[5], Jakob Knudsen[9], Charles Mbogo[10,11], Fredros O. Okumu[12,13,14], Lorenz von Seidlein[15], Daniel J. Weiss[5], Steve W. Lindsay[16], Peter W. Gething[5] & Samir Bhatt[5,17]

[1]Department of Disease Control, London School of Hygiene & Tropical Medicine, London, UK. [2]RTI International, Washington, DC, USA. [3]Division of Epidemiology and Public Health, School of Medicine, University of Nottingham, Nottingham, UK. [4]United Nations Human Settlements Programme, Geneva, Switzerland. [5]Big Data Institute, Nuffield Department of Medicine, University of Oxford, Oxford, UK. [6]Health Metrics and Measurement Cluster, World Health Organization, Geneva, Switzerland. [7]UCL Institute for Environmental Design and Engineering (IEDE), University College London, London, UK. [8]Department of Mathematics and Data Science Institute, Imperial College London, London, UK. [9]School of Architecture, The Royal Danish Academy of Fine Arts, Copenhagen, Denmark. [10]Kenya Medical Research Institute, Kilifi, Kenya. [11]KEMRI-Wellcome Trust Research Program, Nairobi, Kenya. [12]Environmental Health and Ecological Sciences Department, Ifakara Health Institute, Ifakara, Tanzania. [13]School of Public Health, Faculty of Health Sciences, University of the Witwatersrand, Johannesburg, South Africa. [14]Institute of Biodiversity, Animal Health and Comparative Medicine, University of Glasgow, Glasgow, UK. [15]Mahidol-Oxford Tropical Medicine Research Unit (MORU), Faculty of Tropical Medicine, Mahidol University, Bangkok, Thailand. [16]Department of Biosciences, Durham University, Durham, UK. [17]Department of Infectious Disease Epidemiology, Imperial College London, London, UK. *e-mail: lucy.tusting@lshtm.ac.uk

Supplementary Information:
Mapping changes in housing in sub-Saharan Africa from 2000 to 2015

Table of Contents

Classification of house type....2

Drinking-water source and sanitation 2

Living area....2

Housing durability 2

Supplementary Tables (S1 to S7)....3

Table S1. Predicted prevalence of houses built with finished materials and improved housing in 2000 and 2015 by country 3

Table S2. Characteristics of 51 surveys included in the meta-analysis of wealth, education and age 6

Table S3. World Health Organization Joint Monitoring Programme classification of drinking-water source and sanitation facilities [1] 8

Table S4. Availability of georeferenced survey data on house construction materials and house type 9

Table S5. Classification of house construction materials in 96 national surveys 12

Table S6. Model performance of pixel level cross validation 23

Table S7. Model performance of administrative division 1 cross validation 23

References....24

Classification of house type

We used the same categorization of house type as the Millennium Development Goals and Sustainable Development Goals (Extended Data Table 1). We considered unimproved housing to have at least one of four characteristics: (1) unimproved water supply (as defined by the World Health Organisation Joint Monitoring Programme (WHO-JMP)[1]); (2) unimproved sanitation (as defined by WHO-JMP[1]), (3) more than three people per bedroom and (4) house made of natural or unfinished material. Details of these criteria are below. Following United Nations (UN) protocol, we excluded a fifth characteristic of unimproved housing from our definition (insecurity of tenure) due to a lack of internationally comparable data.[2]

Drinking-water source and sanitation

We used the standard drinking-water and sanitation categories developed by the World Health Organization and United Nations International Children's Fund Joint Monitoring Programme (Table S3).[1] An 'improved' drinking-water source is considered one that adequately protects the source from outside contamination, particularly faecal matter. An 'improved' sanitation facility is one that hygienically separates human excreta from human contact.

Living area

Aligning with United Nations (UN) criterion, a house was considered to provide a sufficient living area for the household members if not more than three people share the same sleeping room.[2]

Housing durability

The UN estimation procedure uses the building materials for the roof, walls and/or floor to measure the durability of housing.[2] DHS and MIS surveys classify wall, roof and floor materials as 'natural', 'rudimentary' or 'finished'.[3] While definitions vary by country and year (Table S5), finished wall materials typically include cement, stone with lime or cement, burnt bricks, cement blocks, covered adobe and wood planks or shingles and natural or rudimentary wall materials typically include cane, palm, dirt, bamboo or stone with mud, uncovered adobe, plywood, cardboard and reused wood planks. Finished roof materials typically include metal, wood, cement fibre, ceramic tiles, cement and roofing shingles and natural or rudimentary roof materials typically include thatch, palm leaf, sod, rustic matting, bamboo, wood planks and cardboard. Finished floor materials include parquet or polished wood, vinyl or asphalt strips, ceramic tiles, cement and carpet and natural or rudimentary floor materials include earth, sand, dung, wood planks, palm and bamboo.[3]

In our study, houses were classified as 'built with finished materials' if at least two out of three of the wall, roof and floor materials were finished, and as 'built with natural or unfinished materials' if this criterion was not met. In contrast to the DHS and MIS methods, and aligning with previous studies on housing and health,[4,5] we considered wood and wood shingles to be natural materials (not finished) that may contain openings more permeable to mosquitoes and sand-flies, relevant for the transmission of vector-borne diseases. We also considered sheet metal always to be a finished material.[6]

Supplementary Tables (S1 to S7)

Table S1. Predicted prevalence of houses built with finished materials and improved housing in 2000 and 2015 by country

Country	Indicator	Predicted prevalence (95% confidence intervals)					
		2000			2015		
		National	Urban	Rural	National	Urban	Rural
Angola	Finished materials	28.6 (26.8-30.0)	85.6 (81.2-88.9)	15.8 (14.4-17.5)	47.7 (45.9-50.3)	92.9 (91.1-94.6)	31.1 (28.7-34.2)
	Improved housing	10.3 (8.4-11.7)	29.9 (23.5-37.1)	5.8 (5.2-6.6)	21.2 (18.1-24.3)	44.4 (36.0-54.3)	12.5 (11.1-14.1)
Benin	Finished materials	52.5 (46.2-58.1)	85.1 (81.5-87.7)	47.0 (40.2-53.1)	73.9 (68.1-79.3)	92.7 (90.8-94.1)	70.0 (63.1-76.2)
	Improved housing	10.8 (9.1-13.4)	24.0 (20.1-29.9)	8.6 (7.2-10.8)	25.9 (23.1-29.5)	49.5 (44.5-55.1)	20.9 (18.4-24.5)
Botswana	Finished materials	64.5 (57.6-69.6)	88.1 (85.0-90.9)	59.7 (51.8-65.0)	85.0 (81.9-87.8)	95.0 (93.4-96.1)	82.9 (79.3-86.1)
	Improved housing	30.3 (25.0-34.9)	44.4 (36.1-51.3)	27.1 (22.7-31.6)	51.8 (47.2-55.6)	69.9 (63.5-74.5)	48.1 (43.0-52.0)
Burkina Faso	Finished materials	31.6 (26.5-36.9)	83.8 (78.1-87.7)	26.8 (21.2-32.6)	55.5 (50.2-63.8)	93.2 (90.5-94.5)	49.8 (43.8-59.2)
	Improved housing	9.5 (7.7-11.4)	41.3 (31.8-49.7)	6.6 (5.4-8.4)	27.9 (25.0-30.9)	71.4 (66.4-76.3)	21.3 (18.1-24.5)
Burundi	Finished materials	16.7 (12.3-20.1)	64.7 (56.8-71.3)	15.1 (10.8-18.5)	43.3 (38.2-48.7)	87.1 (84.2-89.4)	41.3 (35.9-46.9)
	Improved housing	7.9 (6.5-9.9)	31.1 (24.3-40.0)	7.2 (5.8-9.0)	22.0 (18.4-25.9)	65.5 (58.8-72.2)	20.1 (16.6-23.9)
Cameroon	Finished materials	36.7 (33.1-40.5)	79.9 (75.3-83.6)	29.7 (25.9-33.8)	64.1 (60.4-67.9)	92.1 (90.4-93.6)	58.4 (54.1-62.8)
	Improved housing	14.0 (12.0-16.3)	39.6 (33.3-46.8)	9.8 (8.1-11.6)	34.9 (32.0-37.6)	69.6 (65.1-73.6)	27.5 (25.0-30.6)
Central African Republic	Finished materials	17.1 (14.4-20.5)	61.2 (50.6-69.0)	12.0 (10.0-15.2)	28.9 (25.2-33.2)	79.4 (71.8-83.9)	24.4 (20.5-28.9)
	Improved housing	4.4 (3.2-6.0)	18.5 (12.5-27.0)	2.8 (2.0-3.7)	13.7 (11.3-15.9)	44.4 (34.8-54.2)	10.8 (8.5-12.9)
Congo	Finished materials	44.7 (39.4-48.1)	80.6 (73.4-86.0)	33.3 (28.7-36.9)	78.8 (75.5-81.6)	90.8 (87.3-93.2)	71.2 (67.3-74.9)
	Improved housing	14.8 (11.9-18.0)	27.6 (20.6-35.2)	10.7 (9.1-13.0)	38.6 (32.9-43.8)	47.6 (40.3-55.1)	32.9 (28.8-37.6)
Côte d'Ivoire	Finished materials	57.8 (50.5-63.8)	92.7 (89.1-94.4)	52.4 (44.4-59.3)	79.7 (75.4-84.5)	97.5 (96.6-98.1)	76.0 (70.9-81.8)
	Improved housing	16.0 (12.8-19.3)	35.1 (28.9-43.6)	13.0 (10.3-15.8)	37.7 (34.5-41.9)	62.7 (54.9-67.6)	32.5 (29.0-37.6)
Democratic Republic of the Congo	Finished materials	15.5 (14.0-17.0)	69.4 (64.1-74.0)	9.3 (8.0-10.6)	27.8 (26.1-29.5)	84.9 (82.4-86.8)	19.4 (17.5-21.1)
	Improved housing	6.1 (5.5-7.0)	27.0 (22.9-32.5)	3.8 (3.3-4.2)	12.3 (10.8-13.4)	42.8 (35.9-49.2)	7.7 (6.8-8.5)
Equatorial Guinea	Finished materials	34.3 (28.7-42.3)	86.9 (81.1-90.3)	31.6 (25.8-39.9)	67.9 (60.1-72.6)	94.5 (92.3-95.9)	66.5 (58.4-71.4)
	Improved housing	9.6 (7.7-11.7)	39.2 (29.4-50.5)	8.1 (6.3-10.2)	23.2 (19.1-28.2)	65.2 (54.9-74.2)	21.2 (16.9-26.1)
Eritrea	Finished materials	5.7 (4.3-8.0)	34.6 (26.6-43.8)	5.1 (3.9-7.4)	15.6 (12.7-19.2)	63.9 (56.2-70.5)	14.4 (11.5-18.0)
	Improved housing	4.1 (3.3-5.4)	19.4 (14.8-25.4)	3.8 (3.0-5.1)	9.0 (7.2-11.5)	39.7 (33.3-48.7)	8.2 (6.4-10.7)

Country	Indicator	Predicted prevalence (95% confidence intervals)					
		2000			2015		
		National	Urban	Rural	National	Urban	Rural
Ethiopia	Finished materials	6.1 (5.1-7.0)	54.1 (44.5-60.5)	4.5 (3.7-5.3)	14.7 (13.1-16.7)	77.3 (73.1-80.4)	12.0 (10.4-14.1)
	Improved housing	4.2 (3.6-4.9)	29.7 (23.4-36.0)	3.4 (2.8-4.0)	7.2 (6.5-8.4)	49.8 (43.4-55.8)	5.4 (4.7-6.4)
Gabon	Finished materials	55.6 (50.5-60.0)	86.1 (81.4-89.5)	45.0 (38.9-50.7)	81.2 (78.1-84.0)	94.7 (92.7-96.2)	75.4 (71.4-79.0)
	Improved housing	21.4 (18.4-25.7)	39.1 (32.1-48.7)	15.4 (13.0-18.3)	46.6 (43.4-50.0)	67.6 (61.6-73.7)	37.4 (35.0-41.1)
Gambia	Finished materials	46.9 (42.5-52.2)	82.4 (75.7-87.2)	33.6 (28.9-39.5)	77.3 (73.3-82.0)	92.9 (88.8-95.0)	70.0 (64.7-76.5)
	Improved housing	21.3 (17.5-26.0)	42.6 (31.7-52.7)	13.6 (11.1-16.2)	42.6 (37.8-48.2)	63.5 (52.7-71.9)	33.0 (28.1-38.4)
Ghana	Finished materials	70.1 (63.9-74.4)	90.6 (87.3-93.2)	65.9 (58.4-70.6)	88.5 (85.7-90.8)	97.1 (96.0-97.8)	86.2 (82.6-89.1)
	Improved housing	19.3 (15.5-22.4)	32.1 (27.1-37.7)	16.4 (12.8-20.0)	36.1 (31.9-41.8)	44.4 (37.5-51.2)	33.8 (29.3-39.9)
Guinea	Finished materials	30.2 (25.9-34.5)	81.7 (76.4-85.0)	23.9 (19.7-28.6)	61.1 (55.9-67.0)	94.1 (93.2-95.1)	56.5 (50.6-63.1)
	Improved housing	11.1 (9.8-13.1)	35.6 (29.8-43.6)	8.2 (6.9-10.0)	29.3 (26.1-32.9)	62.5 (57.2-67.8)	24.6 (21.3-28.2)
Guinea-Bissau	Finished materials	30.7 (25.4-35.3)	77.7 (67.8-84.0)	24.8 (19.5-29.6)	61.9 (53.4-69.1)	92.4 (88.4-95.0)	56.4 (47.2-65.3)
	Improved housing	10.6 (8.6-13.2)	32.0 (24.2-45.5)	7.9 (6.5-9.7)	26.4 (22.8-30.2)	57.9 (44.9-67.2)	21.0 (17.7-25.2)
Kenya	Finished materials	30.2 (25.5-34.5)	79.4 (73.9-83.7)	25.7 (21.0-30.2)	55.9 (52.0-61.0)	92.9 (91.9-93.8)	51.5 (47.3-57.3)
	Improved housing	13.9 (12.0-15.7)	41.0 (35.5-47.0)	11.3 (9.7-13.3)	27.8 (24.9-30.2)	58.7 (53.4-63.0)	24.2 (21.1-26.8)
Liberia	Finished materials	31.5 (27.3-35.5)	75.8 (65.9-82.9)	23.5 (20.1-27.2)	52.7 (48.1-57.0)	92.9 (90.5-94.7)	45.1 (39.9-50.0)
	Improved housing	9.8 (7.8-11.5)	26.5 (18.6-31.9)	6.8 (5.7-7.9)	20.9 (18.1-24.7)	45.1 (35.6-52.7)	16.4 (13.9-20.0)
Madagascar	Finished materials	19.7 (17.3-22.5)	71.5 (65.8-76.7)	15.4 (13.1-18.3)	37.0 (33.2-41.7)	85.2 (83.0-87.6)	32.1 (28.1-37.6)
	Improved housing	4.5 (3.8-5.6)	19.4 (14.1-27.9)	3.3 (2.9-3.9)	9.9 (8.6-11.5)	35.4 (28.5-45.6)	7.3 (6.4-8.3)
Malawi	Finished materials	23.4 (19.5-27.0)	73.1 (68.8-77.7)	20.8 (16.7-24.5)	46.5 (41.6-53.3)	87.6 (85.0-90.3)	44.1 (38.9-51.1)
	Improved housing	7.9 (6.6-9.1)	28.9 (24.1-34.4)	6.6 (5.6-8.0)	13.6 (11.7-15.7)	46.4 (39.2-53.2)	11.7 (10.0-13.6)
Mali	Finished materials	21.4 (18.1-23.9)	72.8 (65.1-76.6)	16.8 (13.8-19.6)	37.8 (34.5-43.9)	85.9 (83.4-88.7)	32.1 (28.6-38.7)
	Improved housing	9.0 (7.4-10.4)	35.3 (28.3-39.9)	6.8 (5.6-8.0)	23.0 (20.6-25.6)	64.2 (57.7-69.1)	18.3 (16.1-20.8)
Mozambique	Finished materials	24.2 (21.4-27.0)	71.9 (67.7-75.7)	19.5 (16.8-22.4)	37.2 (33.6-40.8)	82.7 (78.5-85.7)	32.3 (28.5-36.4)
	Improved housing	9.0 (7.7-10.0)	24.9 (21.8-29.7)	7.5 (6.3-8.5)	14.6 (13.3-16.3)	41.3 (35.7-47.1)	11.8 (10.5-13.5)
Namibia	Finished materials	42.2 (37.8-46.7)	85.5 (80.3-89.0)	36.2 (31.6-41.1)	69.8 (64.7-74.4)	93.7 (89.8-95.6)	65.8 (60.1-70.8)
	Improved housing	21.9 (19.2-25.3)	49.3 (37.7-60.1)	18.2 (16.1-21.3)	40.0 (36.0-43.5)	71.4 (65.7-76.4)	34.4 (30.1-38.3)
Nigeria	Finished materials	49.3 (45.5-52.8)	86.1 (82.9-88.3)	42.1 (37.7-45.9)	71.2 (67.5-74.0)	94.9 (93.9-95.5)	65.1 (60.7-68.6)
	Improved housing	13.0 (11.3-14.7)	29.8 (26.3-33.9)	9.6 (8.3-11.1)	30.4 (27.8-33.4)	52.7 (48.0-56.4)	24.7 (22.3-28.0)

Country	Indicator	Predicted prevalence (95% confidence intervals)					
		2000			2015		
		National	Urban	Rural	National	Urban	Rural
Rwanda	Finished materials	18.5 (14.5-22.3)	62.0 (54.8-68.7)	16.3 (12.2-20.0)	43.0 (39.2-48.2)	83.6 (79.6-86.3)	39.9 (35.9-45.6)
	Improved housing	10.1 (8.5-12.1)	37.6 (29.7-45.6)	8.6 (7.1-10.5)	25.3 (22.3-28.8)	68.5 (60.8-73.4)	22.3 (19.0-25.9)
Senegal	Finished materials	47.2 (42.3-51.1)	87.4 (83.3-89.7)	35.8 (30.6-41.0)	74.4 (69.4-79.9)	95.3 (93.9-96.3)	68.0 (61.7-75.3)
	Improved housing	23.1 (19.7-26.5)	46.2 (39.4-53.6)	16.5 (14.3-19.4)	43.9 (39.4-48.0)	67.9 (61.4-72.2)	36.7 (31.7-41.1)
Sierra Leone	Finished materials	28.0 (22.9-32.1)	71.8 (63.3-77.3)	22.9 (18.2-27.1)	57.7 (52.3-64.1)	93.2 (91.1-94.7)	53.1 (47.1-60.1)
	Improved housing	11.3 (9.3-13.2)	32.9 (25.2-39.6)	8.7 (7.2-10.4)	25.0 (22.2-28.9)	52.1 (43.4-59.9)	21.6 (18.9-25.3)
Somalia	Finished materials	13.4 (10.9-16.5)	36.8 (26.5-46.4)	11.0 (8.9-14.4)	27.7 (23.2-32.4)	62.0 (50.1-73.8)	23.4 (19.0-27.9)
	Improved housing	10.6 (9.0-12.6)	33.2 (25.7-40.2)	8.2 (6.9-10.1)	16.7 (13.4-19.6)	45.1 (35.2-54.4)	12.8 (10.5-15.6)
South Sudan	Finished materials	4.1 (3.3-5.5)	35.5 (28.7-44.9)	3.6 (2.8-5.1)	9.0 (7.3-11.2)	62.6 (55.2-70.6)	8.0 (6.3-10.3)
	Improved housing	3.1 (2.4-3.7)	22.8 (17.8-29.1)	2.7 (2.2-3.4)	6.4 (5.2-8.0)	45.6 (37.7-55.0)	5.7 (4.5-7.3)
Sudan	Finished materials	16.1 (13.9-18.9)	54.2 (46.9-60.4)	12.3 (10.3-15.0)	26.3 (22.8-30.2)	73.4 (69.1-78.2)	21.6 (17.8-25.4)
	Improved housing	10.7 (9.2-12.5)	30.6 (25.9-35.6)	8.8 (7.3-10.7)	17.4 (15.0-20.6)	51.4 (45.3-56.3)	14.0 (11.6-17.0)
Swaziland	Finished materials	66.5 (59.9-72.4)	91.9 (89.1-94.5)	65.3 (58.2-71.3)	82.2 (77.6-84.9)	96.1 (95.0-96.9)	81.5 (76.7-84.3)
	Improved housing	19.7 (16.3-23.4)	37.2 (27.8-47.5)	18.9 (15.5-22.3)	38.3 (32.8-43.7)	59.3 (49.5-68.8)	37.4 (31.7-42.7)
Togo	Finished materials	55.0 (47.4-60.1)	84.7 (80.5-88.9)	49.8 (41.5-55.3)	79.6 (73.5-84.3)	95.8 (94.5-96.7)	76.3 (69.2-81.9)
	Improved housing	12.1 (10.1-14.3)	28.6 (22.7-34.2)	9.2 (7.6-11.3)	25.8 (22.6-30.2)	52.6 (46.7-57.9)	20.6 (17.6-24.9)
Uganda	Finished materials	20.3 (17.3-24.1)	75.6 (67.0-79.9)	16.5 (13.3-20.5)	42.7 (38.2-48.4)	91.3 (88.9-92.7)	37.8 (32.8-44.0)
	Improved housing	7.9 (6.6-9.1)	33.2 (26.6-40.7)	6.0 (4.8-7.2)	19.7 (17.0-21.9)	59.5 (51.7-65.4)	15.6 (13.0-18.0)
United Republic of Tanzania	Finished materials	21.9 (18.5-25.2)	75.5 (70.8-78.7)	17.0 (13.8-20.5)	55.5 (49.8-60.8)	92.3 (90.9-93.6)	51.3 (44.9-57.0)
	Improved housing	8.2 (7.2-9.4)	28.6 (23.7-34.2)	6.4 (5.5-7.4)	21.7 (19.6-24.3)	53.2 (47.9-57.3)	18.2 (16.0-20.9)
Zambia	Finished materials	35.8 (32.7-38.7)	89.9 (86.2-92.0)	28.1 (24.7-31.1)	58.0 (53.8-61.8)	96.2 (95.3-97.0)	51.4 (46.6-55.8)
	Improved housing	14.0 (11.9-15.7)	40.4 (33.8-46.5)	10.1 (8.6-11.6)	28.0 (25.2-30.1)	64.5 (58.2-69.2)	21.7 (19.2-23.6)
Zimbabwe	Finished materials	53.9 (47.4-58.4)	93.1 (91.1-94.8)	50.2 (43.1-55.1)	75.8 (69.8-79.6)	96.7 (95.6-97.6)	73.4 (66.8-77.6)
	Improved housing	29.6 (25.4-32.5)	61.9 (55.4-67.7)	26.6 (22.5-29.5)	44.4 (40.6-49.6)	76.1 (71.1-79.7)	40.8 (36.9-46.5)

Table S2. Characteristics of 51 surveys included in the meta-analysis of wealth, education and age

Survey	Total households[1]	% households in upper 75% wealth quartile	% household head completed secondary education	% household head aged >55 years	% improved house
Benin 1996	4320	24.3	13.7	27.2	6.9
Benin 2006	17113	23.9	19.2	22.1	9.6
Benin 2012	16785	23.2	20.6	25.3	17.6
Burkina Faso 2010	14264	24.9	10.9	25.6	22.0
Burundi 2010	8448	22.8	11.7	20.9	17.2
Burundi 2012	4824	22.1	11.2	21.1	19.8
Cameroon 2011	13918	17.5	39.1	27.4	35.4
Comoros 2012	4338	23.6	32.5	24.4	23.9
Congo (Brazzaville) 2005	5654	24.7	63.0	21.0	15.0
Congo (Brazzaville) 2011	11360	24.8	58.6	22.4	14.2
Congo Democratic Republic 2013	18025	24.7	52.8	21.7	7.0
Cote d'Ivoire 2012	9390	23.8	21.9	26.0	28.9
Ethiopia 2011	16556	24.4	14.0	23.3	11.5
Gabon 2000	5891	12.4	42.0	31.3	26.1
Gabon 2012	9399	19.3	50.2	33.1	29.9
Gambia 2013	6046	24.3	25.4	33.7	38.6
Ghana 2008	11585	23.8	55.9	24.4	36.7
Ghana 2014	11805	23.3	57.7	25.7	31.2
Guinea 2012	7054	25.0	19.3	35.0	29.1
Kenya 2008	8962	23.7	34.5	23.3	26.3
Kenya 2014	35998	24.2	33.5	24.1	23.6
Lesotho 2009	7839	25.8	23.5	37.7	18.5
Lesotho 2014	9215	24.8	28.3	38.0	37.9
Liberia 2013	9278	24.1	40.4	20.7	12.7
Madagascar 2008	17251	23.8	29.0	19.7	4.5
Malawi 2010	24467	23.5	20.0	24.1	6.0
Mali 2012	9960	24.3	14.1	27.5	19.2
Mozambique 2011	13225	24.6	18.0	21.7	10.7
Namibia 2006	8839	25.1	44.6	27.2	34.0
Namibia 2013	9620	20.6	54.0	26.7	38.0
Niger 2012	10557	24.4	10.4	26.3	9.4
Nigeria 2008	32792	20.0	38.2	24.4	21.4
Nigeria 2010	5698	24.3	38.7	25.9	19.1
Nigeria 2013	37252	25.1	42.7	26.3	25.4
Rwanda 2010	12412	24.9	11.7	21.8	29.2
Rwanda 2015	12620	22.2	13.6	23.3	25.3

Survey	Total households[1]	% households in upper 75% wealth quartile	% household head completed secondary education	% household head aged >55 years	% improved house
Senegal 2010	7658	21.7	11.6	39.6	26.1
Senegal 2012	4068	21.3	12.9	38.1	28.1
Senegal 2014	4088	19.3	11.5	38.4	28.1
Sierra Leone 2008	7047	18.5	26.1	27.8	18.6
Sierra Leone 2013	12376	21.2	24.6	24.5	19.2
Swaziland 2006	4743	24.8	44.7	26.8	32.3
Tanzania 2010	9548	23.3	14.0	27.1	12.8
Tanzania 2012	9878	20.2	13.0	26.6	21.2
Togo 1998	7339	22.3	24.6	26.1	11.6
Togo 2013	9417	23.9	36.6	24.4	23.5
Uganda 2006	8663	18.5	22.6	21.2	10.4
Zambia 2007	7024	24.0	37.9	21.4	15.4
Zambia 2013	15563	25.0	44.4	21.2	22.0
Zimbabwe 2005	9112	22.6	48.9	25.7	42.9
Zimbabwe 2010	9608	24.7	53.5	24.6	43.2

[1]Complete cases only

Table S3. World Health Organization Joint Monitoring Programme classification of drinking-water source and sanitation facilities [1]

	Drinking-water source	Sanitation
**Improved**	Piped water into dwelling	Flush toilet
	Piped water to yard/plot	Piped sewer system
	Public tap or standpipe	Septic tank
	Tubewell or borehole	Flush/pour flush to pit latrine
	Protected dug well	Ventilated improved pit latrine (VIP)
	Protected spring	Pit latrine with slab
	Rainwater	Composting toilet
		Special case
**Unimproved**	Unprotected spring	Flush/pour flush to elsewhere
	Unprotected dug well	Pit latrine without slab
	Cart with small tank/drum	Bucket
	Tanker-truck	Hanging toilet or hanging latrine
	Surface water	Shared sanitation
	Bottled water	No facilities or bush or field

Table S4. Availability of georeferenced survey data on house construction materials and house type

Survey	Type	No. households sampled	Variables available or calculable				
			Floor material	Wall material	Roof material	House construction materials	House type[1]
Angola 2006	MIS	2599	Yes	-	-	-	-
Angola 2011	MIS	8391	Yes	Yes	Yes	Yes	Yes
Benin 1996	DHS	4499	Yes	Yes	Yes	Yes	Yes
Benin 2001	DHS	5769	Yes	Yes	Yes	Yes	-
Benin 2012	DHS	17422	Yes	Yes	Yes	Yes	Yes
Burkina Faso 1993	DHS	5143	Yes	-	-	-	-
Burkina Faso 1999	DHS	4812	Yes	-	-	-	-
Burkina Faso 2003	DHS	9097	Yes	-	-	-	-
Burkina Faso 2010	DHS	14424	Yes	Yes	Yes	Yes	Yes
Burkina Faso 2014	MIS	6448	Yes	Yes	Yes	Yes	Yes
Burundi 2010	DHS	8596	Yes	Yes	Yes	Yes	Yes
Burundi 2012	MIS	4866	Yes	Yes	Yes	Yes	Yes
Cameroon 1991	DHS	3538	Yes	-	-	-	-
Cameroon 2004	DHS	10462	Yes	-	-	-	-
Cameroon 2011	DHS	14214	Yes	Yes	Yes	Yes	Yes
Central African Republic 1994	DHS	5551	Yes	Yes	Yes	Yes	Yes
Comoros 2012	DHS	4482	Yes	Yes	Yes	Yes	Yes
Congo, Democratic Republic 2007	DHS	8886	Yes	-	Yes	-	-
Congo, Democratic Republic 2013	DHS	18171	Yes	Yes	Yes	Yes	Yes
Cote d'Ivoire 1994	DHS	5935	Yes	-	-	-	-
Cote d'Ivoire 1998	DHS	2122	Yes	-	-	-	-
Cote d'Ivoire 2012	DHS	9686	Yes	Yes	Yes	Yes	Yes
Ethiopia 2000	DHS	14072	Yes	-	Yes	-	-
Ethiopia 2005	DHS	13721	Yes	Yes	Yes	Yes	-
Ethiopia 2011	DHS	16702	Yes	Yes	Yes	Yes	Yes
Gabon 2012	DHS	9755	Yes	Yes	Yes	Yes	Yes
Ghana 1993	DHS	5822	Yes	-	-	-	-
Ghana 1998	DHS	6003	Yes	-	-	-	-
Ghana 2003	DHS	6251	Yes	-	-	-	-
Ghana 2008	DHS	11778	Yes	Yes	Yes	Yes	Yes
Ghana 2014	DHS	11835	Yes	Yes	Yes	Yes	Yes
Guinea 1999	DHS	5090	Yes	-	-	-	-
Guinea 2005	DHS	6282	Yes	-	-	-	-
Guinea 2012	DHS	7109	Yes	Yes	Yes	Yes	Yes
Kenya 2003	DHS	8561	Yes	-	Yes	-	-
Kenya 2008	DHS	9057	Yes	Yes	Yes	Yes	Yes
Kenya 2014	DHS	36430	Yes	Yes	Yes	Yes	Yes
Kenya 2015	MIS	6481	Yes	Yes	Yes	Yes	Yes
Lesotho 2004	DHS	8592	Yes	-	-	-	-
Lesotho 2009	DHS	9396	Yes	Yes	Yes	Yes	Yes

Survey	Type	No. households sampled	Variables available or calculable				
			Floor material	Wall material	Roof material	House construction materials	House type[1]
Lesotho 2014	DHS	9402	Yes	Yes	Yes	Yes	Yes
Liberia 2007	DHS	6824	Yes	Yes	Yes	Yes	Yes
Liberia 2009	MIS	4162	Yes	Yes	Yes	Yes	Yes
Liberia 2011	MIS	4162	Yes	Yes	Yes	Yes	Yes
Liberia 2013	DHS	9333	Yes	Yes	Yes	Yes	Yes
Madagascar 1997	DHS	7171	Yes	-	-	-	-
Madagascar 2008	DHS	17857	Yes	Yes	Yes	Yes	Yes
Madagascar 2011	MIS	8094	Yes	Yes	Yes	Yes	Yes
Madagascar 2013	MIS	8574	Yes	Yes	Yes	Yes	Yes
Malawi 2000	DHS	14213	Yes	-	-	-	-
Malawi 2004	DHS	13664	Yes	-	-	-	-
Malawi 2010	DHS	24825	Yes	Yes	Yes	Yes	Yes
Malawi 2012	MIS	3404	Yes	Yes	Yes	Yes	Yes
Malawi 2014	MIS	3405	Yes	Yes	Yes	Yes	Yes
Mali 1996	DHS	8716	Yes	-	-	-	-
Mali 2001	DHS	12331	Yes	-	-	-	-
Mali 2006	DHS	12998	Yes	-	-	-	-
Mali 2012	DHS	10107	Yes	Yes	Yes	Yes	Yes
Mozambique 2011	DHS	13919	Yes	Yes	Yes	Yes	Yes
Namibia 2000	DHS	6392	Yes	-	-	-	-
Namibia 2006	DHS	9200	Yes	Yes	Yes	Yes	Yes
Namibia 2013	DHS	9849	Yes	Yes	Yes	Yes	Yes
Niger 1992	DHS	5242	Yes	-	Yes	-	-
Niger 1998	DHS	5928	Yes	-	-	-	-
Nigeria 2003	DHS	7225	Yes	-	-	-	-
Nigeria 2008	DHS	34070	Yes	Yes	Yes	Yes	Yes
Nigeria 2010	MIS	5895	Yes	Yes	Yes	Yes	Yes
Nigeria 2013	DHS	38522	Yes	Yes	Yes	Yes	Yes
Rwanda 2005	DHS	10272	Yes	-	-	-	-
Rwanda 2008	DHS	7377	Yes	-	-	-	-
Rwanda 2010	DHS	12540	Yes	Yes	Yes	Yes	Yes
Rwanda 2015	DHS	12698	Yes	Yes	Yes	Yes	Yes
Senegal 1993	DHS	3528	Yes	-	-	-	-
Senegal 1997	DHS	4772	Yes	-	-	-	-
Senegal 2005	DHS	7412	Yes	-	-	-	-
Senegal 2008	MIS	10651	Yes	Yes	Yes	Yes	Yes
Senegal 2010	DHS	7904	Yes	Yes	Yes	Yes	Yes
Senegal 2012	DHS	4177	Yes	Yes	Yes	Yes	Yes
Sierra Leone 2008	DHS	7284	Yes	Yes	Yes	Yes	Yes
Sierra Leone 2013	DHS	12629	Yes	Yes	Yes	Yes	Yes
Swaziland 2006	DHS	4843	Yes	Yes	Yes	Yes	Yes
Tanzania 1999	DHS	3615	Yes	-	-	-	-
Tanzania 2010	DHS	9623	Yes	Yes	Yes	Yes	Yes

Survey	Type	No. households sampled	Variables available or calculable				
			Floor material	Wall material	Roof material	House construction materials	House type[1]
Tanzania 2012	AIS	10040	Yes	Yes	Yes	Yes	Yes
Togo 1998	DHS	7517	Yes	Yes	Yes	Yes	Yes
Togo 2013	DHS	9549	Yes	Yes	Yes	Yes	Yes
Uganda 2000	DHS	7885	Yes	Yes	Yes	Yes	-
Uganda 2006	DHS	8870	Yes	Yes	Yes	Yes	Yes
Uganda 2009	MIS	4421	Yes	Yes	Yes	Yes	Yes
Uganda 2011	DHS	21478	Yes	Yes	Yes	Yes	Yes
Uganda 2014	MIS	5345	Yes	Yes	Yes	Yes	Yes
Zambia 2007	DHS	7164	Yes	Yes	Yes	Yes	Yes
Zambia 2013	DHS	15920	Yes	Yes	Yes	Yes	Yes
Zimbabwe 1999	DHS	6369	Yes	-	-	-	-
Zimbabwe 2005	DHS	9285	Yes	Yes	Yes	Yes	Yes
Zimbabwe 2010	DHS	9756	Yes	Yes	Yes	Yes	Yes
Total surveys			**96**	**62**	**66**	**62**	**59**

[1] House type was determined using the following four variables: (1) type of water supply, (2) type of sanitation facility, (3) number of household members per bedroom, (4) house construction materials.

Table S5. Classification of house construction materials in 96 national surveys

Survey	Survey type	Number of households	Roof		Walls		Floor	
			Natural or unfinished	Finished	Natural or unfinished	Finished	Natural or unfinished	Finished
Angola 2006	MIS	2599	n/a	n/a	n/a	n/a	Earth; sand; dung; wood	Parquet; polished wood; ceramic tiles; mosaic; cement; carpet
Angola 2011	MIS	8391	Palm; bamboo; mat; wood planks; tarpaulin; plastic; other	Zinc; metal; asbestos; ceramic tiles; concrete; cement	Straw; mats; cardboard; plastic; sticks and mud; clay blocks; cane; palm; trunks; used wood; wood planks; other	Concrete; stone blocks; bricks	Clay; sand; dung; tablets; wood planks; other	Parquet; polished wood; ceramic; mosaic; tiles; cement; carpet
Benin 1996	DHS	4499	Earth; straw; other	Metal; tile; cement	Earth; bamboo; other	Brick; stone; semi-durable	Earth; wood; other	Cement
Benin 2001	DHS	5769	Earth; leaves; wood planks; palm branches; other	Metal; tile; cement	Earth; stone; wood planks; palm branches; other	Bricks; mixed brick	Earth; sand; dung; wood planks; palm; bamboo; other	Parquet; polished wood; vinyl; cement
Benin 2012	DHS	17422	No roof; thatch; palm leaf; sod; rustic mat; palm; bamboo; wood planks; cardboard; wood; roofing shingles; other	Metal; cement fibre; ceramic tiles; cement	No walls; cane; palm; trunks; dirt; bamboo with mud; stone with mud; uncovered adobe; plywood; cardboard; reused wood; wood planks or shingles; other	Cement; stone with lime or cement; bricks; cement blocks; covered adobe	Earth; sand; dung; wood planks; palm, bamboo; other	Parquet; polished wood; vinyl; asphalt strips; ceramic tiles; cement; carpet
Burkina Faso 1993	DHS	5143	n/a	n/a	n/a	n/a	Earth; sand; dung; other	Parquet; polished wood; vinyl; asphalt strips; ceramic tiles; cement; carpet
Burkina Faso 1999	DHS	4812	n/a	n/a	n/a	n/a	Sand; dirt; other	Vinyl; tile; cement; carpet
Burkina Faso 2003	DHS	9097	n/a	n/a	n/a	n/a	Earth; sand; dung; wood planks; palm; bamboo; other	Parquet; polished wood; vinyl; asphalt strips; ceramic tiles; cement; carpet
Burkina Faso 2010	DHS	14424	No roof; thatch; palm leaf; sod; rustic mat; palm; bamboo; wood planks; wood; roofing shingles; other	Metal; zinc with cement fibre; tiles; slate; cement	No walls; cane; palm; trunk; dirt; bamboo with mud; stone with mud; uncovered adobe; wood planks or shingles; other	Cement; stone with lime or cement; bricks; cement blocks; covered adobe	Earth; sand; dung; wood planks; palm; bamboo; other	Parquet or polished wood; ceramic tiles; cement; carpet
Burkina Faso 2014	MIS	6448	Thatch; palm leaf; wood; other	Metal	Cane; palm; trunks; dirt; bamboo with mud; stone with mud; uncovered adobe; wood planks or shingles; other	Cement; stone with lime or cement; bricks; cement blocks; covered adobe	Earth; sand; dung; other	Ceramic tiles; cement

Survey	Survey type	Number of households	Roof		Walls		Floor	
			**Natural or unfinished**	**Finished**	**Natural or unfinished**	**Finished**	**Natural or unfinished**	**Finished**
Burundi 2010	DHS	8596	No roof; thatch; palm leaf; sod; rustic mat; bamboo; wood planks; cardboard; shingles; other	Metal; tiles; zinc with cement fibre; tiles; slate; cement	No walls; cane; palm; trunks; dirt; bamboo with mud; stone with mud; uncovered adobe; reused wood; wood planks or shingles; other	Cement; stone with lime or cement; bricks; cement blocks; covered adobe	Earth; sand; dung; wood planks; palm; other	Parquet; polished wood; vinyl; asphalt strips; ceramic tiles; cement; carpet
Burundi 2012	MIS	4866	No roof; thatch; palm leaf; sod; palm; bamboo; wood; roofing shingles; other	Metal; ceramic tiles; cement	No walls; bamboo; palm; trunk; dirt; bamboo with mud; stone with mud; mud bricks; reused wood; wood planks or shingles	Cement; stone with lime or cement; bricks; cement blocks; covered adobe	Earth; sand; dung; other	Ceramic tiles; cement; carpet
Cameroon 1991	DHS	3538	n/a	n/a	n/a	n/a	Earth; wood; other	Cement; tile
Cameroon 2004	DHS	10462	n/a	n/a	n/a	n/a	Earth; sand; wood planks; palm; bamboo; other	Parquet; polished wood; vinyl; asphalt strips; ceramic tiles; cement; carpet
Cameroon 2011	DHS	14214	No roof; thatch; palm leaf; sod; straw; rustic mat; palm; bamboo; wood planks; cardboard; wood; roofing shingles; other	Metal; cement fibre; ceramic tiles; cement	No walls; cane; palm; trunks; dirt; bamboo with mud; stone with mud; uncovered adobe; plywood; cardboard; reused wood; wood planks or shingles; other	Cement; stone with lime or cement; bricks; cement blocks; covered adobe	Earth; sand; dung; wood planks; palm; bamboo; other	Parquet; polished wood; vinyl; asphalt strips; ceramic tiles; cement; carpet
Central African Republic 1994	DHS	5551	Bamboo; straw; other	Cement; tin	Earth; unbaked bricks; planks; other	Cement; finished bricks	Earth; planks	Tiles; cement
Comoros 2012	DHS	4482	No roof; thatch; palm leaf; sod; rustic mat; palm; bamboo; wood; cardboard; roofing shingles; other	Metal; ceramic tiles; cement	No walls; cane; palm; trunks; dirt; palm leaves; bamboo with mud; stone with mud; uncovered adobe; plywood; cardboard; reused wood; shingles; other	Cement; stone with lime or cement; bricks; cement blocks; covered adobe; metal	Earth; sand; dung; wood planks; palm; bamboo; other	Parquet; polished wood; ceramic tiles; cement; carpet
Congo Democratic Republic 2007	DHS	8886	Palm; mat; bamboo; planks; other	Concrete slabs; metal; tile	n/a	n/a	Earth; wood; other	Cement; tile
Congo Democratic Republic 2013	DHS	18171	No roof; thatch; palm leaf; earth; rustic mat; palm; bamboo; wood planks; wood; shingles; other	Metal; zinc or cement fibre; tiles; slate; cement	No walls; cane; palm; trunks; dirt; bamboo with mud; stone with mud; uncovered adobe; bamboo; reused wood; wood; wood planks or shingles; other	Cement; stone with lime or cement; bricks; cement blocks; covered adobe	Earth; sand; dung; wood planks; palm; bamboo; other	Vinyl; asphalt strips; ceramic tiles; cement; carpet
Cote d'Ivoire 1994	DHS	5935	n/a	n/a	n/a	n/a	Earth; sand; dung; wood planks; palm; bamboo	Parquet; polished wood; vinyl; asphalt strips; ceramic tiles; cement

Survey	Survey type	Number of households	Roof		Walls		Floor	
			Natural or unfinished	Finished	Natural or unfinished	Finished	Natural or unfinished	Finished
Cote d'Ivoire 1998	DHS	2122	n/a	n/a	n/a	n/a	Earth; sand; wood; palm; bamboo	Tiles; cement; other finished
Cote d'Ivoire 2012	DHS	9686	No roof; thatch; palm leaf; sod; rustic mat; palm; bamboo; wood planks; wood; cardboard; roofing shingles; other	Metal; ceramic tiles; cement	No walls; cane; palm; trunk; dirt; bamboo or stone with mud; uncovered adobe; plywood; cardboard; reused wood; wood planks or shingles; other	Cement; stone with lime or cement; bricks; cement blocks; covered adobe	Earth; sand; dung; wood planks; palm; bamboo; other	Parquet; polished wood; vinyl; asphalt strips; ceramic tiles; cement; carpet
Ethiopia 2000	DHS	14072	Wood; mulch; thatch; reed; bamboo; plastic sheet; other	Iron; cement; concrete	n/a	n/a	Earth; sand; dung; wood planks; reed; bamboo; other	Parquet; polished wood; vinyl sheets; tiles; cement; brick; carpet
Ethiopia 2005	DHS	13721	Thatch; rustic mat; plastic; reed; bamboo; wood planks; wood; shingles; other	Corrugated iron; cement fibre; cement; concrete	No walls; bamboo; wood; stone with mud; uncovered adobe; plywood; carton; shingles; wood and grass; other	Cement; stone with lime or cement; bricks; cement blocks; covered adobe	Earth; sand; dung; wood planks; reed; bamboo; other	Parquet; polished wood; vinyl; ceramic tiles; cement; bricks; carpet
Ethiopia 2011	DHS	16702	No roof; thatch; mud; rustic mat; plastic sheet; reed; bamboo; cardboard; wood; other	Metal; asbestos; cement fibre; concrete; cement	No walls; cane; trunk; dirt; bamboo with mud; stone with mud; uncovered adobe; plywood; cardboard; reused wood; wood planks or shingles; other	Cement; stone with lime or cement; bricks; cement blocks; covered adobe	Earth; sand; dung; wood planks; palm; bamboo; other	Parquet; polished wood; vinyl; asphalt strips; ceramic tiles; cement; carpet
Gabon 2012	DHS	9755	Plastic; cardboard; bark; straw; palm; other	Metal; tiles; slate; concrete	Plastic; cardboard; bark; straw; palm; dirt; mud bricks; planks; other	Metal; semi-durable; cement	Earth; sand; wood planks; other	Parquet; polished wood; linoleum; ceramic tiles; cement; carpet
Ghana 1993	DHS	5822	n/a	n/a	n/a	n/a	Earth; sand; dung; wood planks; palm; bamboo	Parquet; polished wood; vinyl; asphalt strips; ceramic tiles; cement; carpet; terrazzo
Ghana 1998	DHS	6003	n/a	n/a	n/a	n/a	Earth; mud; dung; wood planks; palm; bamboo	Linoleum; ceramic tiles; cement; carpet; terrazzo
Ghana 2003	DHS	6251	n/a	n/a	n/a	n/a	Earth; sand; mud; dung; wood planks; palm; bamboo; other	Parquet; polished wood; linoleum; ceramic tiles; cement; carpet; terrazzo
Ghana 2008	DHS	11778	No roof; thatch; palm; rustic mat; wood planks; wood; shingles; other	Metal; cement fibre; tiles; cement; asbestos; slate	No walls; cane; palm; trunks; dirt; bamboo or stone with mud; plywood; reused wood; shingles; other	Cement; stone with lime or cement; bricks; cement blocks; covered adobe	Earth; sand; dung; wood planks; palm; bamboo; other	Parquet; polished wood; ceramic tiles; terrazzo; cement; carpet; linoleum

Survey	Survey type	Number of households	Roof		Walls		Floor	
			**Natural or unfinished**	**Finished**	**Natural or unfinished**	**Finished**	**Natural or unfinished**	**Finished**
Ghana 2014	DHS	11835	No roof; thatch; palm leaf; rustic mat; palm; bamboo; wood planks; cardboard; wood; roofing shingles; other	Metal; cement fibre; ceramic tiles; cement; asbestos; slate	No walls; cane; palm; trunks; dirt; bamboo with mud; stone with mud; uncovered adobe; plywood; cardboard; reused wood; wood planks or shingles; other	Cement; stone with lime or cement; bricks; cement blocks; covered adobe	Earth; sand; dung; wood planks; other	Parquet; polished wood; vinyl; asphalt strips; ceramic; marble; porcelain; cement; carpet; linoleum
Guinea 1999	DHS	5090	n/a	n/a	n/a	n/a	Earth; sand; wood	Cement; tile; other finished
Guinea 2005	DHS	6282	n/a	n/a	n/a	n/a	Earth; sand; wood planks	Cement; ceramic tiles; other finished
Guinea 2012	DHS	7109	No roof; thatch; palm leaf; rustic mat; wood; other	Metal; zinc or cement fibre; tiles; slate; cement	No walls; cane; palm; trunks; dirt; bamboo, wood or stone with mud; other	Cement; stone with lime or cement; cement blocks	Earth; sand; dung; palm; bamboo; other	Parquet; polished wood; vinyl; asphalt strips; ceramic tiles; cement
Kenya 2003	DHS	8561	Grass; thatch; makuti; tin cans; other	Corrugated iron sheets; asbestos; concrete; tiles	n/a	n/a	Earth; mud; dung; sand; wood planks; palm; bamboo; other	Parquet; polished wood; vinyl; asphalt strips; ceramic tiles; cement; carpet
Kenya 2008	DHS	9057	Thatch; palm; dung; mud; other	Iron; tin; asbestos sheets; concrete; tiles	No walls; cane; palm; trunks; dirt; dung; bamboo or stone with mud; uncovered adobe; plywood; cardboard; reused wood; shingles; other	Metal; cement; stone with lime or cement; bricks; cement blocks; covered adobe	Earth; sand; dung; wood planks; palm; bamboo; other	Parquet; polished wood; vinyl; asphalt strips; ceramic tiles; cement; carpet
Kenya 2014	DHS	36430	No roof; thatch; grass; makuti; dung; mud; sod; other	Metal; asbestos; concrete; tiles	No walls; cane; palm; trunks; dung; mud; sod; grass; bamboo or stone with mud; uncovered adobe; plywood; cardboard; reused wood; wood planks or shingles; other	Metal; cement; stone with lime or cement; bricks; cement blocks; covered adobe	Earth; sand; dung; wood planks; palm; bamboo; other	Parquet; polished wood; vinyl; asphalt strips; ceramic tiles; cement; carpet
Kenya 2015	MIS	6481	No roof; thatch; grass; makuti; dung; mud; sod; other	Iron sheets; tin cans; asbestos sheet; concrete; tiles	No walls; cane; palm; trunks; dung; mud; sod; bamboo with mud; stone with mud; uncovered adobe; plywood; cardboard; reused wood; wood planks or shingles; other	Iron sheets; cement; stone with lime or cement; bricks; cement blocks; covered adobe	Earth; sand; dung; wood planks; other	Parquet or polished wood; vinyl; asphalt; ceramic tiles; cement; carpet
Lesotho 2004	DHS	8592	n/a	n/a	n/a	n/a	Mud; earth; dung; wood planks; other	Parquet; polished wood; brick tiles; tiles; cement; carpet; vinyl

Survey	Survey type	Number of households	Roof		Walls		Floor	
			**Natural or unfinished**	**Finished**	**Natural or unfinished**	**Finished**	**Natural or unfinished**	**Finished**
Lesotho 2009	DHS	9396	Thatch; sod; cardboard; shingles; other	Metal; asbestos; ceramic tiles; cement	Cane; sod; stone; plywood; cardboard; reused wood; shingles; other	Cement; stone with lime or cement; bricks; cement blocks	Mud; earth; wood planks; other	Parquet; polished wood; vinyl; asphalt strips; ceramic tiles; brick tiles; cement; carpet
Lesotho 2014	DHS	9402	Thatch; grass; sod; wood planks; cardboard; wood; roofing shingles	Metal; asbestos or cement fibre; tiles; cement	Cane; trunks; sod; stone with mud; plywood; cardboard; reused wood; wood planks or shingles; other	Cement; stone with lime or cement; bricks; cement blocks	Earth; mud; dung; wood planks; other	Parquet; polished wood; vinyl; ceramic tiles; cement; carpet
Liberia 2007	DHS	6824	Thatch; palm; bamboo; matting; wood planks; tarpaulin; wood; other	Zinc; metal; ceramic tiles; concrete; cement; asbestos sheets	Mud and sticks; cane; palm; straw; thatch; mud bricks; plywood; reused wood; cardboard; plastic; wood planks; other	Metal; cement or stone block; brick	Earth; sand; mud; wood planks; other	Parquet; polished wood; floor mat; linoleum; ceramic tiles; concrete; cement; carpet
Liberia 2009	MIS	4162	Thatch; palm leaf; palm; bamboo; mats; tarpaulin; plastic; other	Zinc; metal; ceramic tiles; concrete; cement; asbestos	Mud and sticks; cane; palm; trunks; straw; thatch mats; mud bricks; cardboard; plastic; wood planks or shingles; other	Cement or stone blocks; bricks; zinc	Earth; sand; mud; wood planks; other	Parquet; polished wood; floor mat; linoleum; ceramic tiles; concrete; cement; carpet
Liberia 2011	MIS	4162	Thatch; palm leaf; palm; bamboo; wood planks; tarpaulin; plastic; wood	Metal; ceramic tiles; concrete; cement; asbestos sheets	Mud and sticks; cane; palm; trunk; straw or thatched mats; mud bricks, plywood; cardboard; plastic; reused wood; wood planks or shingles; other	Cement; stone blocks; bricks; zinc	Earth; sand; mud; wood planks; other	Parquet; polished wood; floor mat; linoleum; ceramic tiles; concrete; cement; carpet
Liberia 2013	DHS	9333	Thatch; palm leaf; rustic mat; palm; bamboo; wood planks; plastic; wood	Metal; ceramic tiles; concrete; asbestos	Mud and sticks; cane; palm; trunks; straw; thatch mats; mud bricks; plywood; cardboard; plastic; reused wood; wood planks or shingles; other	Metal; cement; stone blocks; bricks	Earth; sand; mud; wood planks; other	Parquet; polished wood; floor mat; linoleum; ceramic tiles; terrazzo; concrete; carpet
Madagascar 1997	DHS	7171	n/a	n/a	n/a	n/a	Earth; sand; dung; wood planks; palm; bamboo; other	Parquet; polished wood; vinyl; asphalt strips; ceramic tiles; cement; carpet
Madagascar 2008	DHS	17857	No roof; thatch; palm; sod; rustic mat; palm; bamboo; wood planks; cardboard; wood; shingles; other	Metal; cement fibre; ceramic tiles; cement	No walls; cane; palm; trunks; dirt; bamboo or stone with mud; uncovered adobe; plywood; cardboard; reused wood; wood planks; shingles; other	Cement; stone with lime or cement; bricks; cement blocks	Earth; sand; dung; wood planks; palm; bamboo; mat; other	Parquet; polished wood; vinyl; ceramic tiles; cement; carpet

Survey	Survey type	Number of households	Roof		Walls		Floor	
			Natural or unfinished	Finished	Natural or unfinished	Finished	Natural or unfinished	Finished
Madagascar 2011	MIS	8094	No roof; thatch; palm leaf; sod; rustic mat; palm with bamboo; wood planks; cardboard; wood; roofing shingles; other	Sheet metal; zinc or cement fibre; ceramic tiles; cement	No walls; cane; palm; trunks; dirt; bamboo with mud; stone with mud; plywood; cardboard; reused wood; wood planks or shingles; other	Cement; stone with lime or cement; bricks; cement blocks; sheet metal; tiles; ceramic	Mud; earth; dung; wood planks; palm; bamboo; mat; other	Parquet; polished wood; vinyl; asphalt strips; ceramic tiles; cement; carpet
Madagascar 2013	MIS	8574	No roof; thatch; palm; leaf; sod; rustic mat; palm; bamboo; wood planks; cardboard; wood; roofing shingles; other	Sheet metal; zinc or cement fibre; ceramic tiles; cement	No walls; cane; palm; trunks; dirt; bamboo with mud; stone with mud; plywood; cardboard; reused wood; wood planks or shingles; other	Cement; stone with lime or cement; bricks; cement blocks	Mud; earth; dung; wood planks; palm; bamboo; mat; other	Parquet; polished wood; vinyl; asphalt strips; ceramic tiles; cement; carpet
Malawi 2000	DHS	14213	n/a	n/a	n/a	n/a	Earth; sand; dung; wood planks; broken bricks	Parquet; polished wood; vinyl; asphalt; ceramic tiles; cement; brick
Malawi 2004	DHS	13664	n/a	n/a	n/a	n/a	Earth; sand; dung; wood planks; palm; bamboo; other	Parquet; polished wood; vinyl; linoleum; ceramic tiles; cement; carpet
Malawi 2010	DHS	24825	No roof; thatch; palm leaf; rustic mat; palm; bamboo; grass; wood planks; cardboard; wood; roofing shingles; other	Metal; cement fibre; ceramic tiles; cement	No walls; cane; palm; trunks; dirt; bamboo with mud; stone with mud; plywood; reused wood; wood planks or shingles; other	Cement; stone with lime or cement; bricks; cement blocks	Earth; sand; dung; wood planks; palm; bamboo; broken bricks; other	Parquet; polished wood; vinyl; asphalt strips; ceramic tiles; cement; carpet
Malawi 2012	MIS	3404	No roof; thatch; palm leaf; palm, bamboo; grass; wood planks; cardboard; wood; roofing shingles; other	Iron sheets; cement fibre; ceramic tiles; cement	No walls; cane; palm; trunks; dirt; bamboo or tree trunks with mud; stone with mud; plywood; cardboard; reused wood; other	Cement; stone with lime or cement; burnt bricks; unburnt bricks; cement blocks	Earth; sand; dung; broken bricks	Parquet; polished wood; vinyl; asphalt strips; ceramic tiles; cement; carpet
Malawi 2014	MIS	3405	No roof; thatch; palm leaf; rustic mat; palm; bamboo; grass; wood planks; wood; roofing shingles; other	Iron sheets; cement fibre; ceramic tiles; cement	No walls; cane; palm; trunks; dirt; bamboo with mud; stone with mud; plywood; cardboard; wood planks or shingles; other	Cement; stone with lime or cement; burnt bricks; unburnt bricks; cement blocks	Earth; sand; dung; wood planks; broken bricks; other	Parquet; polished wood; vinyl; asphalt strips; ceramic tiles; cement; carpet
Mali 1996	DHS	8716	n/a	n/a	n/a	n/a	Earth; sand; dung; palm; bamboo	Parquet; polished wood; vinyl; asphalt strips; ceramic tiles; cement; carpet
Mali 2001	DHS	12331	n/a	n/a	n/a	n/a	Variable labels not available	Variable labels not available

Survey	Survey type	Number of households	Roof		Walls		Floor	
			Natural or unfinished	Finished	Natural or unfinished	Finished	Natural or unfinished	Finished
Mali 2006	DHS	12998	n/a	n/a	n/a	n/a	Earth; sand; dung; other	Parquet; polished wood; vinyl; asphalt strips; ceramic tiles; cement; carpet
Mali 2012	DHS	10107	No roof; thatch; palm leaf; sod; rustic mat; palm; bamboo; wood planks; cardboard; wood; roofing shingles; other	Metal; cement fibre; ceramic tiles; cement	No walls; cane; palm; trunk; dirt; bamboo with mud; stone with mud; uncovered adobe; plywood; cardboard; reused wood; wood planks or shingle; other	Cement; stone with lime or cement; bricks; cement blocks; covered adobe	Earth; sand; dung; wood planks; palm; bamboo; other	Parquet; polished wood; vinyl; asphalt strips; ceramic tiles; cement; carpet
Mozambique 2011	DHS	13919	No roof; grass; thatch; palm; other	Metal; cement fibre; ceramic tiles; concrete slab; cement	No walls; cane; palm; trunk; tin/cardboard/paper; sticks; shells (casca); wood or metal planks; adobe; other	Bricks; cement blocks	Earth; wood planks; adobe; other	Parquet; polished wood; tiles; bricks; cement
Namibia 2000	DHS	6392	n/a	n/a	n/a	n/a	Earth; sand; dung; wood planks; palm; other	Vinyl; linoleum; ceramic tiles; cement; carpet
Namibia 2006	DHS	9200	No roof; thatch; palm leaf; sod; rustic mat; palm; bamboo; wood planks; cardboard; wood; shingles; other	Metal; cement fibre; ceramic tiles; cement; corrugated asbestos	No walls; palm; trunks; dirt; dung; bamboo with mud; stone with mud; uncovered adobe; plywood; cardboard; reused wood; shingles; other	Metal; corrugated asbestos; cement; stone with lime or cement; bricks; cement blocks; covered adobe	Earth; sand; dung; wood planks; palm; bamboo; other	Parquet; polished wood; vinyl; asphalt strips; ceramic tiles; cement; carpet
Namibia 2013	DHS	9849	No roof; thatch; palm leaf; sod; rustic mat; palm; bamboo; wood; cardboard; sticks with mud; plastic; roofing shingles; other	Metal; cement fibre; ceramic or brick tiles; cement; asbestos	No walls; cane; palm; trunks; dirt; bamboo, sticks or stones with mud; uncovered adobe; plywood; cardboard; reused wood; wood planks or shingles; other	Cement; stone with lime or cement; bricks; cement blocks; covered adobe; corrugated iron; tin	Earth; sand; dung; mud; clay; wood planks; palm; bamboo; other	Parquet; polished wood; vinyl; asphalt strips; ceramic tiles; cement; carpet
Niger 1992	DHS	5242	Variable labels not available	Variable labels not available	n/a	n/a	Earth; sand	Parquet; polished wood; vinyl; asphalt strips; ceramic tiles; cement; carpet
Niger 1998	DHS	5928	n/a	n/a	n/a	n/a	Earth; sand; dung; other	Ceramic tiles; cement; carpet
Nigeria 2003	DHS	7225	n/a	n/a	n/a	n/a	Earth; sand; dung; wood planks; palm; bamboo	Parquet; polished wood; vinyl; asphalt strips; ceramic tiles; cement; carpet

Survey	Survey type	Number of households	Roof		Walls		Floor	
			Natural or unfinished	Finished	Natural or unfinished	Finished	Natural or unfinished	Finished
Nigeria 2008	DHS	34070	No roof; thatch; palm leaf; sod; rustic mat; palm; bamboo; wood planks; wood; cardboard; other	Metal; cement fibre; ceramic tiles; cement	No walls; cane; palm; trunk; dirt; bamboo with mud; stone with mud; uncovered adobe; plywood; cardboard; wood planks or shingles; other	Cement; stone with lime or cement; bricks; cement blocks; covered adobe	Earth; sand; dung; wood planks; palm; bamboo; other	Parquet; polished wood; vinyl; asphalt strips; ceramic tiles; cement; carpet
Nigeria 2010	MIS	5895	Thatch; palm leaf; palm; bamboo; mats; wood planks; tarpaulin; plastic; wood	Zinc; metal; ceramic tiles; concrete; cement; asbestos	Mud and sticks; cane; palm; trunk; straw or thatched mats; mud bricks, plywood; reused wood; cardboard; plastic; wood planks or shingles; other	Cement or stone blocks; bricks	Earth; sand; wood planks	Parquet; polished wood; floor mat; linoleum; ceramic tiles; concrete; cement; carpet
Nigeria 2013	DHS	38522	No roof; thatch; palm leaf; rustic mat; palm; bamboo; wood planks; cardboard; wood; shingles; other	Metal; ceramic tiles; cement	No walls; cane; palm; trunks; dirt; bamboo with mud; stone with mud; plywood; cardboard; reused wood; wood planks or shingles; other	Metal; cement; stone with lime or cement; bricks; cement blocks	Earth; sand; dung; wood planks; palm; bamboo; other	Parquet; polished wood; vinyl; asphalt strips; ceramic tiles; cement; carpet
Rwanda 2005	DHS	10272	n/a	n/a	n/a	n/a	Earth; sand; dung; other	Parquet; polished wood; linoleum; ceramic tiles; cement; carpet
Rwanda 2008	DHS	7377	n/a	n/a	n/a	n/a	Earth; sand; dung	Parquet; polished wood; ceramic tiles; cement; carpet
Rwanda 2010	DHS	12540	No roof; thatch; palm leaf; sod; rustic mat; plastic; palm; bamboo; wood; roofing shingles; other	Metal; cement fibre; ceramic tiles; cement	No walls; cane; palm; trunk; dirt; bamboo with mud; stone with mud; uncovered adobe; plywood; reused wood; trunks with mud; wood planks or shingles; other	Cement; stone with lime or cement; bricks; cement blocks; covered adobe	Earth; sand; dung; wood planks; other	Ceramic tiles; cement
Rwanda 2015	DHS	12698	No roof; thatch; palm leaf; sod; rustic mat; plastic; palm; bamboo; wood; roofing shingles	Iron sheets; cement fibre; ceramic tiles; cement	No walls; cane; palm; trunks; dirt; bamboo or stone with mud; uncovered adobe; cardboard; reused wood; wood planks or shingles; other	Cement; stone with lime or cement; bricks; cement blocks; covered adobe	Earth; sand; dung; other	Ceramic tiles; cement; carpet
Senegal 1993	DHS	3528	n/a	n/a	n/a	n/a	Earth; sand; dung; other	Parquet; polished wood; vinyl; asphalt strips; ceramic tiles; cement; carpet
Senegal 1997	DHS	4772	n/a	n/a	n/a	n/a	Earth; sand; dung; other	Parquet; polished wood; vinyl; asphalt strips; ceramic tiles; cement; carpet

Survey	Survey type	Number of households	Roof		Walls		Floor	
			Natural or unfinished	Finished	Natural or unfinished	Finished	Natural or unfinished	Finished
Senegal 2005	DHS	7412	n/a	n/a	n/a	n/a	Earth; sand; dung; palm; bamboo; other	Parquet; polished wood; vinyl; ceramic tiles; cement; carpet
Senegal 2008	MIS	10651	No roof; thatch; palm leaf; sod; rustic mat; palm; bamboo; wood planks; cardboard; wood; roofing shingles; other	Metal; cement fibre; ceramic tiles; cement	No walls; cane; palm; trunks; dirt; bamboo with mud; stone with mud; uncovered adobe; plywood; reused wood; wood planks or shingles; other	Cement; stone with lime or cement; bricks; cement blocks; covered adobe	Earth; sand; dung; wood planks; palm; bamboo; other	Parquet; polished wood; vinyl; asphalt strips; ceramic tiles; cement; carpet
Senegal 2010	DHS	7904	No roof; thatch; palm leaf; sod; rustic mat; palm; bamboo; wood planks; cardboard; wood; roofing shingles; other	Metal; cement fibre; ceramic tiles; cement	No walls; cane; palm; trunks; dirt; bamboo with mud; stone with mud; uncovered adobe; plywood; cardboard; reused wood; wood planks or shingles; other	Cement; stone with lime or cement; bricks; cement blocks; covered adobe	Earth; sand; dung; wood planks; palm; bamboo; other	Parquet; polished wood; vinyl; asphalt strips; ceramic tiles; cement; carpet
Senegal 2012	DHS	4177	No roof; thatch; palm leaf; sod; palm; bamboo; wood planks; cardboard; wood; roofing shingles; other	Metal; cement fibre; ceramic tiles; cement	No walls; cane; palm; trunks; dirt; bamboo with mud; stone with mud; reused wood; wood planks or shingles; other	Cement; stone with lime or cement; bricks; cement blocks; covered adobe	Earth; sand; dung; wood planks; palm; bamboo; other	Parquet; polished wood; vinyl; asphalt strips; ceramic tiles; cement; carpet
Sierra Leone 2008	DHS	7284	No roof; thatch; palm leaf; sod; rustic mat; palm; bamboo; wood planks; cardboard; tarpaulin; wood; roofing shingles; other	Metal; cement fibre; ceramic tiles; cement; asbestos	No walls; cane; palm; trunk; dirt; mud bricks; bamboo with mud; stone with mud; uncovered adobe; plywood; cardboard; reused wood; clay blocks; tarpaulin; wood planks or shingles; other	Corrugated iron sheet; cement; stone with lime or cement; bricks; cement blocks; covered adobe	Earth; sand; dung; stone; wood planks; palm; bamboo; other	Parquet; polished wood; vinyl; asphalt strips; ceramic tiles; cement; carpet
Sierra Leone 2013	DHS	12629	No roof; thatch; palm leaf; sod; rustic mat; palm; bamboo; wood planks; cardboard; tarpaulin; wood; shingles; other	Metal; cement fibre; ceramic tiles; cement; asbestos	No walls; cane; palm; trunks; dirt; bamboo or stone with mud; uncovered adobe; plywood; cardboard; reused wood; shingles; other	Metal; cement; stone with lime or cement; bricks; cement blocks; covered adobe	Earth; sand; dung; wood planks; palm; bamboo; other	Parquet; polished wood; vinyl; asphalt strips; ceramic tiles; cement; carpet
Swaziland 2006	DHS	4843	Grass; wood planks; other	Corrugated iron; asbestos; tiles; slate; concrete	Cane; palm; trunks; mud; bamboo with mud; stone with mud; plywood; carton; reused wood; wood shingles; other	Cement; stone with lime/cement; bricks; cement blocks; mud blocks	Earth; sand; dung; wood planks; other	Parquet; polished wood; vinyl; asphalt strips; ceramic tiles; cement; carpet
Tanzania 1999	DHS	3615	n/a	n/a	n/a	n/a	Earth; sand; wood planks; other	Parquet; polished wood; ceramic tiles; cement

Survey	Survey type	Number of households	Roof		Walls		Floor	
			Natural or unfinished	Finished	Natural or unfinished	Finished	Natural or unfinished	Finished
Tanzania 2010	DHS	9623	Grass; thatch; mud; other	Iron sheets; tiles; concrete; asbestos	Grass; poles and mud; sun-dried bricks; baked bricks; wood; other	Cement blocks; stone	Earth; sand; dung; wood planks; bamboo; other	Parquet; polished wood; vinyl; asphalt strips; ceramic tiles; cement; carpet
Tanzania 2012	AIS	10040	Grass; thatch; mud; other	Iron sheets; tiles; concrete; asbestos	Palm; trunks; bamboo; mud; bamboo or stones with mud; plywood; cardboard; reused wood; wood planks; other	Concrete; stone with lime or cement; sun-dried bricks; baked bricks; cement blocks	Earth; sand; dung; wood planks; timber; palm; bamboo; other	Parquet; polished wood; vinyl; asphalt strips; ceramic tiles; terrazzo; cement; carpet
Togo 1998	DHS	7517	Earth; paving; straw; other	Metal	Earth; rock; bamboo; planks; other	Half solid	Earth; wood; other	Marble; cement
Togo 2013	DHS	9549	No roof; thatch; palm leaf; sod; palm; bamboo; wood planks; cardboard; straw; wood; roofing shingles; other	Metal; cement fibre; ceramic tiles; cement	No walls; cane; palm; trunk; dirt; bamboo with mud; stone with mud; uncovered adobe; plywood; cardboard; reused wood; wood planks or shingles; other	Cement; stone with lime or cement; bricks; cement blocks; covered adobe	Earth; sand; dung; palm; bamboo; other	Parquet; polished wood; vinyl; asphalt strips; ceramic tiles; cement; carpet
Uganda 2000	DHS	7885	Thatched; other	Iron sheets; asbestos; tiles; tin; cement	Thatched; mud and pole; unburnt bricks; burnt bricks with mud; timber	Burnt bricks with cement; cement blocks; stone	Earth; sand; dung; other	Parquet; polished wood; vinyl; asphalt strips; ceramic tiles; cement
Uganda 2006	DHS	8870	Thatch; mud; wood; planks; other	Iron sheets; asbestos; tiles; tin; cement	Thatched; straw; mud and poles; unburnt bricks; unburnt bricks and poles; burnt bricks with mud; timber; other	Cement blocks; stone; burnt bricks with cement	Earth; sand; dung; other	Parquet; polished wood; mosaic; tiles; bricks; cement; stone
Uganda 2009	MIS	4421	Thatched; mud; wood; planks; other	Iron sheets; asbestos; tiles; tin; cement	Thatched; straw; mud and poles; unburnt bricks; unburnt bricks with plaster; burnt bricks with mud; timber; other	Cement blocks; stone; burnt bricks with cement	Earth; sand; dung; other	Mosaic; tiles; bricks; cement; stones
Uganda 2011	DHS	21478	Thatch; mud; wood planks; other	Iron sheets; asbestos; tiles; tin; cement	Thatch; straw; mud and poles; unburnt bricks; unburnt bricks and plaster; burnt bricks with mud; timber; other	Cement blocks; stone; burnt bricks with cement	Earth; sand; dung; other	Parquet; polished wood; mosaic; tiles; bricks; cement; stones
Uganda 2014	MIS	5345	Thatched; mud; palm; wood planks; wood; roofing shingles; other	Tin; iron sheets; cement fibre; tiles; cement; asbestos	No walls; thatched; straw; dirt; mud and poles; stone with mud; reused wood; unburnt bricks (including with plaster or mud); wood planks or shingles; other	Cement; stone with lime or cement; burnt bricks with cement; cement blocks	Earth; sand; dung; wood planks; other	Parquet; polished wood; mosaic; tile; cement; stones; bricks

Survey	Survey type	Number of households	Roof		Walls		Floor	
			**Natural or unfinished**	**Finished**	**Natural or unfinished**	**Finished**	**Natural or unfinished**	**Finished**
Zambia 2007	DHS	7164	No roof; thatch; palm leaf; rustic mat; palm; wood; shingles; mud tiles; other	Metal sheets; cement fibre; ceramic tiles; cement	No walls; cane; palm; trunks; mud; bamboo; pole with mud; stone with mud; plywood; cardboard; reused wood; shingles; other	Cement; stone with lime or cement; bricks; cement blocks	Earth; sand; dung; palm; bamboo; other	Parquet; polished wood; vinyl; ceramic or terrazzo tile; concrete; cement; carpet
Zambia 2013	DHS	15920	No roof; thatch; palm leaf; rustic mat; palm; bamboo; wood planks; cardboard; wood; roofing shingles; other	Metal; cement fibre; tiles; cement	No walls; cane; palm; trunks; mud; bamboo or stone with mud; plywood; cardboard; reused wood; wood planks; other	Cement; stone with lime or cement; bricks; cement blocks	Earth; sand; dung; wood planks; palm; bamboo; other	Parquet; polished wood; vinyl; asphalt strips; ceramic tiles; terrazzo; cement; carpet
Zimbabwe 1999	DHS	6369	n/a	n/a	n/a	n/a	Earth; sand; dung; wood planks; other	Parquet; polished wood; vinyl; asphalt strips; ceramic tiles; cement; carpet
Zimbabwe 2005	DHS	9285	No roof; thatch; rustic mat; wood planks; wood; other	Metal; asbestos; tiles; cement	Cane; trunks; mud; stone with mud; plywood; carton; reused wood; wood planks; other	Cement; stone with lime or cement; bricks; cement blocks	Earth; sand; dung; rudimentary wood planks; other	Parquet; polished wood; vinyl; asphalt strips; ceramic tiles; cement; carpet
Zimbabwe 2010	DHS	9756	No roof; thatch; rustic mat; wood planks; wood; other	Metal; asbestos; tiles; cement	Cane; mud; stone with mud; plywood; carton; reused wood; wood plank; other	Cement; stone with lime or cement; bricks; cement blocks	Earth; sand; dung; wood planks; other	Parquet; polished wood; vinyl; ceramic tiles; cement; carpet

AIS: AIDS Indicator Survey; DHS: Demographic and Health Survey; MIS: Malaria Indicator Survey

Table S6. Model performance of pixel level cross validation

Spatial aggregation	Error measure	Improved housing	Housing built with finished materials
Pixel level	Mean squared error	0.021	0.033
	Correlation %	81.9%	85.8%
Survey aggregate level	Mean squared error	0.0005	0.0021
	Correlation %	96.2%	97.3%

Table S7. Model performance of administrative division 1 cross validation

Spatial aggregation	Error measure	Improved housing	Housing built with finished materials
Administrative division 1	Mean squared error	0.0051	0.0137
	Correlation %	89.6%	90.8%

References

1. WHO/UNICEF Joint Monitoring Programme. *Drinking-water and sanitation categories for monitoring purposes* (WHO/UNICEF Joint Monitoring Programme, 2016).

2 UN. *Indicators for Monitoring the Millennium Development Goals: 7.10 Proportion of urban population living in slums* (United Nations, 2012).

3 ICF International. *Demographic and Health Surveys Methodology*. (ICF International, 2011).

4 Tusting, L.S. *et al.* Housing improvements and malaria risk in sub-Saharan Africa: a multi-country analysis of survey data. *PLoS Med.* e1002234 (2017).

5 Tusting, L.S *et al.* The evidence for improving housing to reduce malaria: a systematic review and meta-analysis. *Malaria J* **14**, 209 (2015).

6 Bradley, J. *et al.* Reduced prevalence of malaria infection in children living in houses with window screening or closed eaves on Bioko Island, Equatorial Guinea. *PLoS ONE* **8**, e80626 (2013).
