# Supplementary material for: Mapping changes in housing in sub-Saharan Africa from 2000 to 2015
Source: Nature. 2019 Mar 27;568(7752):391–4. doi: 10.1038/s41586-019-1050-5 (PMC6784864; doi:10.1038/s41586-019-1050-5)
Supplement: Supplementary file 2 — Reporting Summary [file 41586_2019_1050_MOESM2_ESM.pdf]

nature research

Corresponding author(s): Lucy Tusting, Samir Bhatt

Last updated by author(s): Dec 11, 2018

Reporting Summary

Nature Research wishes to improve the reproducibility of the work that we publish. This form provides structure for consistency and transparency in reporting. For further information on Nature Research policies, see Authors & Referees and the Editorial Policy Checklist.

Statistics

For all statistical analyses, confirm that the following items are present in the figure legend, table legend, main text, or Methods section.

n/a	Confirmed	
☐	☒	The exact sample size (*n*) for each experimental group/condition, given as a discrete number and unit of measurement
☒	☐	A statement on whether measurements were taken from distinct samples or whether the same sample was measured repeatedly
☐	☒	The statistical test(s) used AND whether they are one- or two-sided<br>*Only common tests should be described solely by name; describe more complex techniques in the Methods section.*
☐	☒	A description of all covariates tested
☒	☐	A description of any assumptions or corrections, such as tests of normality and adjustment for multiple comparisons
☐	☒	A full description of the statistical parameters including central tendency (e.g. means) or other basic estimates (e.g. regression coefficient) AND variation (e.g. standard deviation) or associated estimates of uncertainty (e.g. confidence intervals)
☒	☐	For null hypothesis testing, the test statistic (e.g. *F*, *t*, *r*) with confidence intervals, effect sizes, degrees of freedom and *P* value noted<br>*Give P values as exact values whenever suitable.*
☒	☐	For Bayesian analysis, information on the choice of priors and Markov chain Monte Carlo settings
☐	☒	For hierarchical and complex designs, identification of the appropriate level for tests and full reporting of outcomes
☐	☒	Estimates of effect sizes (e.g. Cohen's *d*, Pearson's *r*), indicating how they were calculated

Our web collection on statistics for biologists contains articles on many of the points above.

Software and code

Policy information about availability of computer code

Data collection	Data were processed in Stata 14 and R 3.4.4
Data analysis	Data analysis was performed in R 3.4.4 using Tensorflow version 1.10 and the Raster package 2.6.7. All software is open source. Code is available via codeshare https://codeshare.io/2pm4Px

For manuscripts utilizing custom algorithms or software that are central to the research but not yet described in published literature, software must be made available to editors/reviewers. We strongly encourage code deposition in a community repository (e.g. GitHub). See the Nature Research guidelines for submitting code & software for further information.

Data

Policy information about availability of data

All manuscripts must include a data availability statement. This statement should provide the following information, where applicable:

- Accession codes, unique identifiers, or web links for publicly available datasets
- A list of figures that have associated raw data
- A description of any restrictions on data availability

All data are available to download free of charge by registered users from the DHS Program. Registration is available at https://dhsprogram.com/data/new-user-registration.cfm and data may be downloaded at https://dhsprogram.com/data/dataset_admin/download-manager.cfm. Full dataset access instructions are available at https://dhsprogram.com/data/Using-DataSets-for-Analysis.cfm.

Field-specific reporting

Please select the one below that is the best fit for your research. If you are not sure, read the appropriate sections before making your selection.

☐ Life sciences ☒ Behavioural & social sciences ☐ Ecological, evolutionary & environmental sciences

For a reference copy of the document with all sections, see nature.com/documents/nr-reporting-summary-flat.pdf

Behavioural & social sciences study design

All studies must disclose on these points even when the disclosure is negative.

Study description	A geostatistical analysis was done to quantify changes in housing in sub-Saharan Africa from 2000 to 2015. Further analyses were done to explore the relationship between housing and socioeconomic factors.
Research sample	Data from the Demographic and Health Survey program were analyzed. We included all available surveys from https://dhsprogram.com/ with data on housing variables and that were georeferenced, a total of 62 national household surveys representing 661,945 unique households in 31 countries.
Sampling strategy	All available surveys and data points were included in the analysis.
Data collection	We performed a secondary analysis of survey data.
Timing	The data analysed were from a series of national household surveys conducted over several decades.
Data exclusions	No data were excluded from the analysis.
Non-participation	n/a
Randomization	n/a

Reporting for specific materials, systems and methods

We require information from authors about some types of materials, experimental systems and methods used in many studies. Here, indicate whether each material, system or method listed is relevant to your study. If you are not sure if a list item applies to your research, read the appropriate section before selecting a response.

Materials & experimental systems

n/a	Involved in the study	
☒	☐	Antibodies
☒	☐	Eukaryotic cell lines
☒	☐	Palaeontology
☒	☐	Animals and other organisms
☒	☐	Human research participants
☒	☐	Clinical data

Methods

n/a	Involved in the study	
☒	☐	ChIP-seq
☒	☐	Flow cytometry
☒	☐	MRI-based neuroimaging
